# Chi3l3 induces oligodendrogenesis in an experimental model of autoimmune neuroinflammation

**DOI:** 10.1038/s41467-018-08140-7

**Published:** 2019-01-15

**Authors:** Sarah C. Starossom, Juliana Campo Garcia, Tim Woelfle, Silvina Romero-Suarez, Marta Olah, Fumihiro Watanabe, Li Cao, Ada Yeste, John J. Tukker, Francisco J. Quintana, Jaime Imitola, Franziska Witzel, Dietmar Schmitz, Markus Morkel, Friedemann Paul, Carmen Infante-Duarte, Samia J. Khoury

**Affiliations:** 1 Institute for Medical Immunology, Charité – Universitätsmedizin Berlin, 13353 Berlin, Germany; 2 Experimental and Clinical Research Center, Max Delbrueck Center for Molecular Medicine and Charité - Universitätsmedizin Berlin, Berlin, Germany; 3 NeuroCure Clinical Research Center, Charité - Universitätsmedizin Berlin, 10117 Berlin, Germany; 4 Center for Neurologic Diseases, Department of Neurology, Brigham and Women’s Hospital, Harvard Medical School, Boston, MA 02115 USA; 5 Laboratory of Neural Stem Cells and Functional Neurogenetics, Department of Neurology-The Ohio State University Wexner Medical Center, Columbus, OH 43210 USA; 6 Neuroscience Research Center (NWFZ), Charité – Universitätsmedizin Berlin, 10117 Berlin, Germany; 7 DZNE-German Center for Neurodegenerative Diseases, Charité – Universitätsmedizin Berlin, 10117 Berlin, Germany; 8 IRI Life Sciences, Institute of Pathology, Computational Modeling in Medicine, Charité– Universitätsmedizin Berlin, 10117 Berlin, Germany; 9 Institute of Pathology, Laboratory of Molecular Tumor Pathology and Systems Biology, Charité – Universitätsmedizin Berlin, 10117 Berlin, Germany; 10 Department of Neurology, Charité - Universitätsmedizin Berlin, 10117 Berlin, Germany; 11 Abu Haidar Neuroscience Institute, American University of Beirut Medical Center, Beirut, Lebanon

## Abstract

In demyelinating diseases including multiple sclerosis (MS), neural stem cells (NSCs) can replace damaged oligodendrocytes if the local microenvironment supports the required differentiation process. Although chitinase-like proteins (CLPs) form part of this microenvironment, their function in this differentiation process is unknown. Here, we demonstrate that murine Chitinase 3-like-3 (Chi3l3/Ym1), human Chi3L1 and Chit1 induce oligodendrogenesis. In mice, Chi3l3 is highly expressed in the subventricular zone, a stem cell niche of the adult brain, and in inflammatory brain lesions during experimental autoimmune encephalomyelitis (EAE). We find that silencing Chi3l3 increases severity of EAE. We present evidence that in NSCs Chi3l3 activates the epidermal growth factor receptor (EGFR), thereby inducing Pyk2-and Erk1/2- dependent expression of a pro-oligodendrogenic transcription factor signature. Our results implicate CLP-EGFR-Pyk2-MEK-ERK as a key intrinsic pathway controlling oligodendrogenesis.

## Abstract (web summary)

Chitinase 3-like-3 (Chi3l3) is expressed in microglia, but its function is not clear. Here the authors show that Chi3l3 is expressed in the subventricular zone in mouse experimental immune encephalitis, which induces oligodendrogenesis.

## Introduction

Oligodendrocytes constitute one of the four principal central nervous system (CNS) cell types—along with neurons, astrocytes, and microglia. Within the CNS, oligodendrocytes form myelin sheaths around axons, a prerequisite for efficient signal conduction. However, oligodendrocytes are highly susceptible to injury owing to their elevated metabolic rate and ATP requirement for the synthesis of myelin membranes^1^. Thus, oligodendrogenesis, i.e., differentiation of oligodendrocytes from neural stem cells (NSCs), is vital for both the developing and the adult CNS, ensuring repair and replenishment of damaged myelin.

In the adult brain, NSCs from the subventricular zone (SVZ), a specialized adult stem cell niche adjacent to the lateral ventricle, contribute to local myelin repair by differentiating into oligodendrocyte precursor cells (OPCs) that migrate to the site of injury and subsequently mature into myelinating oligodendrocytes^2–7^. Activation of endogenous NSCs is not cell-autonomous, but depends on the SVZ microenvironment^3,8,9^, which is shaped by SVZ microglia and infiltrating macrophages by means of cell-to-cell contact and/or soluble immune mediators^10–13^.

In demyelinating diseases, such as multiple sclerosis (MS) and the animal model experimental autoimmune encephalomyelitis (EAE), failure of oligodendrogenesis and remyelination result in chronic demyelination and axon degeneration, causing severe disabilities^14–16^. Thus, understanding the molecular mechanisms that drive oligodendrogenesis is crucial for developing strategies for remyelination.

It is known that the activation state of microglia determines their niche-supporting function^17,18^. In EAE, activated microglia have regeneration-supporting functions during the acute phase of the disease and seem to be nonpermissive for oligodendrogenesis and remyelination during chronic disease^9,18^.

The regeneration-supporting microglia express high levels of chitinase 3-like-3 (Chi3l3, Ym1), a known marker for alternative activation of microglia and macrophages (M2)^19^. Chi3l3 is a member of a family of mammalian chitinase-like proteins (CLPs) that share homology to chitinases of lower organisms but lack enzymatic activity^13,20^. Chi3l3 has been implicated in immunomodulation^21–23^, but its function in the CNS is essentially unknown.

Here, we show that Chi3l3 serves as an activator of the epidermal growth factor receptor (EGFR) and induces fate choice towards the oligodendroglial lineage in NSCs in vitro and in vivo. This effect is accompanied by the upregulation of *Olig1*, *Olig2*, *Sox10*, and downregulation of their negative transcriptional regulators *Id2*, *Id4,* and *Hes5*, and is mediated by induction of the mitogen-activated protein kinase (MAPK) pathway in a Pyk2-dependent fashion. Furthermore, our data point toward a critical role of Chi3l3 in reducing disease severity at onset and during chronic autoimmune demyelination.

## Results

### Dynamic regulation of endogenous Chi3l3 in the adult SVZ

**Fig. 1:**
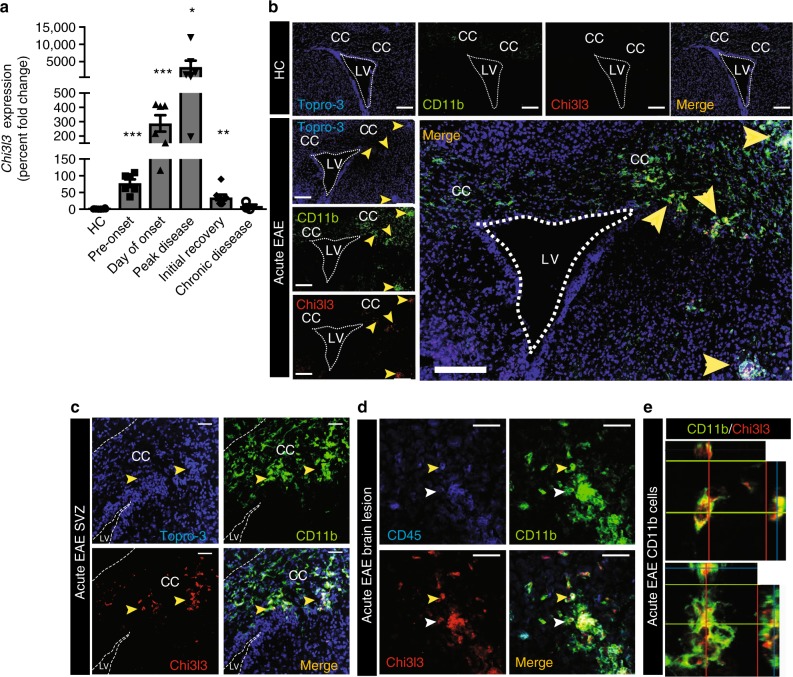
Endogenous *Chi3l3* expression in the adult SVZ during EAE. **a** *Chi3l3* gene expression in the subventricular zone (SVZ) of healthy control mice (HC; *n* = 8), preclinical EAE (6 days post immunization (dpi; *n* = 6), EAE onset (13dpi; *n* = 6), acute EAE (14 dpi; *n* = 6), initial recovery (18 dpi; *n* = 8) and chronic EAE (36 dpi, *n* = 6) measured by qRT-PCR (multiple *t* -tests with Dunn–Bonferroni’s multiple comparison test). **b**–**e** Confocal images of the SVZ of representative healthy controls (HC), acute EAE mice (12–14 dpi). Sections were immunostained for the nuclear marker TO-PRO-3 (blue), Chi3l3 (red) and the microglia/macrophage marker CD11b (green, **b**–**e**) and the lymphocyte common antigen CD45 (blue, **d**). Chi3l3 expression in the SVZ was only detected during acute EAE and was localized to CD11b-positive cells (yellow arrowheads) within the corpus callosum (CC) **c** and lesions **d**, where they co-expressed CD45 at high (yellow arrowhead) and low levels (white arrowhead). Dashed lines mark the wall of the lateral ventricle (LV). Scale bar, 200 μm. **e** Ortho-view images of representative Chi3l3^+^/CD11b^+^ double-positive cells exhibiting round (upper panel) and ramified (lower panel) morphology. (mean ± s.e.m. *p < 0.05; **p < 0.01; ***p < 0.005)

We measured *Chi3l3* gene expression and protein levels in the CNS during relapsing-remitting EAE using quantitative real-time PCR (qRT-PCR) and immunostaining, respectively. Naive mice expressed very modest levels of *Chi3l3* in the SVZ (Fig. 1a). During acute EAE, *Chi3l3* gene expression increased to 78-fold before onset of EAE, 298-fold during onset of clinical EAE signs and to 3471-fold during peak disease. *Chi3l3* gene expression decreased again thereafter to a 35-fold expression during initial recovery and to ninefold expression during chronic EAE, when compared with healthy control mice (Fig. 1a). Chi3l3 protein levels were analyzed during acute and chronic EAE. Immunofluorescent signal was only detectable during acute EAE (Fig. 1b) but not chronic EAE (Supplementary Figure 1A). In agreement with previous reports^19,24^, Chi3l3 protein expression colocalized with the microglia- and macrophage marker CD11b (Fig. 1b–d), but not with CD4^+^-infiltrating T cells (Supplementary Figure 1 B–E). Chi3l3-expressing cells were located periventricularly (Fig. 1c), and in lesions abutting the SVZ. (Fig. 1b, d). Chi3l3^+^ CD11b^+^ cells constituted a heterogeneous population that expressed the activation marker CD45 at high (Fig. 1d, yellow arrowhead) or low levels (Fig. 1d, white arrowhead) and displayed either round (Fig. 1e upper panel) or ramified (Fig. 1e, lower panel) morphology. These results indicate that CD11b^+^ myeloid cells including resident microglia and infiltrating macrophages upregulate Chi3l3 expression during the course of EAE predominantly during acute EAE.

### Chi3l3 induces oligodendrogenesis in vitro

**Fig. 2:**
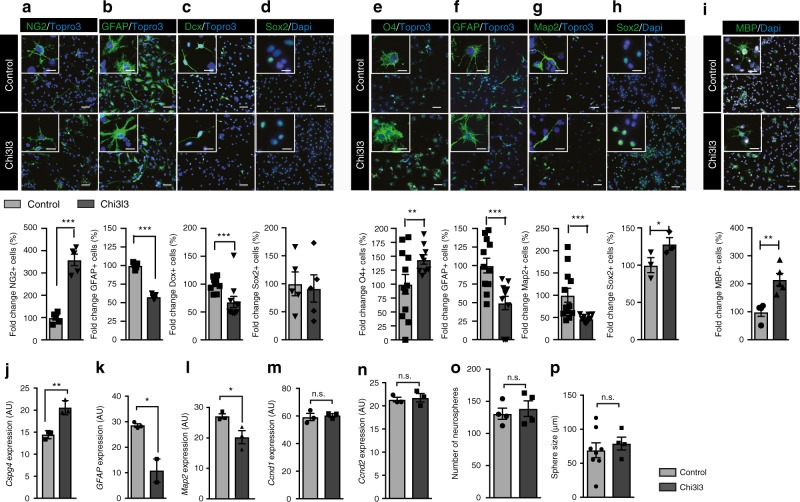
Chi3l3 directly promotes oligodendrogenesis in vitro. Representative confocal image (above) and quantification (below) of neural stem cells (NSCs) cultured in the presence of PBS (control) or Chi3l3 (100 ng/ml) for 3 days **a**–**d**, 5 days **e**–**h**, or primary OPCs cultured in the presence of PBS (control) or Chi3l3 (500 ng/ml) for 7 days **i**. Cells were treated with the nuclear stain TO-PRO-3 (blue) and immunostained for early progenitor markers NG2 (**a**; oligodendrocyte precursor cells, green), GFAP (**b**; astrocytes, green), Dcx (**c**; neuroblasts, green), late progenitor markers O4 (**e**; oligodendrocytes, green), GFAP (**f**; astrocytes, green) and microtubule-associated protein 2 (**g**; Map2, neurons, green), the neural stem cell marker Sox2 (**d**, **h**; green) and the myelin protein MBP (**i**, oligodendrocytes, green). Exposure of differentiating NSCs to Chi3l3 led to significant increase in oligodendrocyte precursor cells and oligodendrocytes, significant decrease in astrocytes, neuroblasts, and neurons and a significant increase in Sox2^+^ neural stem cells. Scale bar, 50 μm. Inserts show representative cells. Scale bar, 20 μm. **j**–**n** Gene expression of *Cspg4* (NG2; **j**), *Gfap* **k**, and *Map2* (**l**; 3 days) and *Ccnd1* and *Ccnd2* (**m**, **n**; 24 h) mRNA in PBS (control) or Chi3l3-treated differentiating NSCs. Values were normalized against *Gapdh* (AU, arbitrary unit; n.s., not significant;). Number **o** and size **p** of neurospheres from NSCs exposed to Chi3l3 or PBS (control). (n.s., not significant; two-tailed Student’s *t* test; data are representative of three independent experiments with *n* = 5 replicates **a**, **c**, **d**, **i**, *n* = 9 (Control) and 10 (Chi3l3) replicates **b**, *n* = 12 (Control) and 9 (Chi3l3) replicates **e**, *n* = 12 replicates **f**, **g**, *n* = 3 replicates **h**, **j**, **l**, **m**, **n**), *n* = 3 (Control) and 2 (Chi3l3) replicates **k**, *n* = 4 replicates **o**, *n* = 8 (control) and four (Chi3l3) replicates **p**). mean ± s.e.m. *p < 0.05; **p < 0.01; ***p < 0.005

We investigated the effects of Chi3l3 on NSCs in vitro, focusing on the differentiation of NSCs into the three neural lineages as well as self-renewal capacity, proliferation, and cell death. We found that NSCs that differentiated in the presence of recombinant Chi3l3 for 3 days (early precursors, Fig. 2a–d) or 5 days (mature cells, Fig. 2e–h) had a higher proportion of NG2-expressing OPCs (Fig. 2a) and O4^+^ oligodendrocytes (Fig. 2e) than control NSCs and a lower proportion of glial fibrillary acidic protein (GFAP)-expressing astrocytes (Fig. 2b, f), doublecortin (Dcx)^+^ neural precursors (Fig. 2c) and Map2^+^ mature neurons (Fig. 2g). The relative increase in Sox2^+^ stem cells detected on day 5 (Fig. 2h) was not owing to proliferation of the stem cells, as levels of BrdU incorporation were below 1% for both vehicle and Chi3l3-stimulated NSCs (Supplementary Figure 2A, B). Furthermore, we examined, whether Chi3l3 also affected OPC maturation towards myelinating oligodendrocytes. Exposure of primary OPCs to recombinant Chi3l3 for 7 days, positively affected the percentage of MBP + mature oligodendrocytes compared with control (Fig. 2i).

The Chi3l3-mediated effects on NSC differentiation were dose-dependent (Supplementary Figure 3). Chi3l3-mediated oligodendrogenesis was accompanied by a significant increase in *Cspg4* (NG2) gene expression and a reduced expression of *Map2* and *Gfap* (Fig. 2j–l).

Chi3l3 did not significantly affect proliferation of NSCs, as measured by *Ccnd1* and *Ccnd2* expression (Fig. 2m, n) and Ki67 staining in total cells (Ki67^+^/DAPI), OPCs (Ki67^+^/NG2^+^ cells), and non-OPCs (Ki67^+^/NG2^−^ cells) (Supplementary Figure 2C). Chi3l3 also did not affect cell survival, as measured by propidium iodide (PI) uptake (Supplementary Figure 2D), or self-renewal capacity, as measured by neurosphere number and size (Fig. 2o, p). Thus, Chi3l3 directs NSC fate choice toward oligodendrogenesis without affecting proliferation, self-renewal capacity, or cell survival.

It is widely accepted that alternatively activated microglia/macrophages are key players in modulating NSC fate choice toward oligodendrogenesis^17^, but the specific mechanisms are unknown. To investigate the role of Chi3l3 in microglia-induced oligodendrogenesis, we knocked down Chi3l3 expression in BV-2 microglia cells and performed medium-transfer assays with NSC cultures. Silencing *Chi3l3* in BV-2 microglial cells led to effective loss of Chi3l3 protein (Supplementary Figure 4A, B) and indeed, prevented microglia-induced oligodendrogenesis (Supplementary Figure 4C).

Taken together, the data show that Chi3l3 affects differentiation of NSCs toward OPCs and mature oligodendrocytes.

### Chi3l3 induces oligodendrogenesis through MAPK signaling

**Fig. 3:**
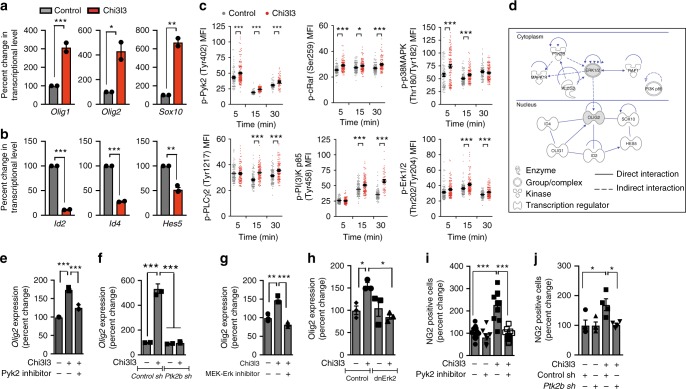
Chi3l3 induces a pro-oligodendrogenic transcriptional and signaling profile. **a**, **b** Gene expression of the pro-oligodendroglial transcription factors *Olig1*, *Olig2,* and *Sox10* after 36 h **a** and negative transcriptional regulators *Id2, Id4,* and *Hes5* after 24 h **b** in differentiating neural stem cells (NSCs) post exposure to Chi3l3 or PBS (control). **c** Mean fluorescence intensity (MFI) quantification of individual NSCs, activated by Chi3l3 or PBS (control), fixed at 5, 15, and 30 min and immunostaining for phosphorylation (p)- specific antibodies to various signaling proteins. **d** Ingenuity pathway analysis showing interconnection between Chi3l3-mediated signaling events (*Ptk2b* (Pyk2), MAPK14 (p38MAPK), PLCG2 (PLCγ2), RAF1 (c-Raf), ERK1/2, PI3K p85 and transcriptional events (*Olig1, Olig2, Sox10, Hes5*, *Id2*, *Id4*). **e**, **f** Gene expression of *Olig2* in differentiating NSCs in the presence of Pyk2 kinase inhibitor PF-431396 **e** or NSCs, transfected with control shLVPs or *Ptk2b* (Pyk2) shLVPs **f** after 36 h of exposure to Chi3l3 or PBS. **g**, **h** Gene expression of *Olig2* in differentiating NSCs in presence of MEK-Erk inhibitor U0126 **g** or NSCs, transfected with LVPs containing GFP (control) or dnErk2-GFP **h** after 36 h of exposure to Chi3l3 or PBS. Values were normalized to housekeeping genes and expressed as percent change to PBS-treated control NSCs. **i** Quantification of NG2^+^ oligodendrocyte precursor cells, derived from NSCs differentiated for 3 days in the presence ( + ) or absence (−) of PF-431396 and Chi3l3 ( + ) or PBS (−). **j** Quantification of NG2^+^ oligodendrocyte precursor cells derived from differentiating NSCs, transfected with control shLVPs or *Ptk2b* shLVPs (Pyk2), and treated with Chi3l3 ( + ) or PBS (−). Pyk2 signaling deficiency significantly reduced Chi3l3-induced oligodendrogenesis. Values are expressed as percent change to the corresponding PBS-treated group. Two-tailed Student’s *t* test was performed for **a**–**c**, one-way ANOVA followed by Turkey’s post hoc test was performed for **e**–**j**; Data are representative of three independent experiments. For *n* numbers please refer the Methods section; mean ± s.e.m. *p < 0.05; **p < 0.01; ***p < 0.005

We measured genetic regulators of oligodendrogenesis in Chi3l3-treated NSCs by quantitative RT-PCR. Genes encoding crucial pro-oligodendrogenic differentiation factors, such as *Olig1*, *Olig2*, and *Sox10*, were significantly upregulated in Chi3l3-treated NSCs compared with control NSCs (Fig. 3a), whereas negative regulators of oligodendrogenesis including *Id2*, *Id4*, and *Hes5*, were significantly downregulated (Fig. 3b).

Using reverse-phase protein arrays, we identified the kinetics (0–30 min) of phosphorylation-patterns of a spectrum of signaling nodes involved in NSC fate choice and inflammation, indicating activation of these kinases^25–27^. Chi3l3 significantly increased the phosphorylation of proteins in the MAPK/phospho-inositol-3-kinase (PI3K) pathways including Pyk2 (Tyr^402^), PLCγ2 (Tyr^1217^), cRaf (Ser^259^), PI(3)K p85 (Tyr^458^), p38MAPK (Thr^180^/Tyr^182^) and Erk1,2 (Thr^202^/Tyr^204^) (Supplementary Figure 5).

This phosphorylation pattern was confirmed at the single-cell level by fluorescence microscopy (Fig. 3c). A significant increase in phosphorylation of Pyk2 (Tyr^402^), cRaf (Ser^259^) and p38MAPK (Thr^180^/Tyr^182^) was detected as early as 5 min post exposure to Chi3l3, followed by phosphorylation of PLCγ2 (Tyr^1217^), PI(3)K p85 (Tyr^458^) and Erk1,2 (Thr^202^/Tyr^204^) at 15 min post exposure. Activation of p38MAPK (Thr^180^/Tyr^182^), occurred only transiently, but phosphorylation of all other kinases analyzed was sustained for 30 min (Fig. 3c).

To link signaling molecules with transcriptional regulators modulated by Chi3l3, we performed bioinformatic pathway analysis using Ingenuity® pathway analysis (IPA). The latter revealed that Pyk2 (*Ptk2b*) was predicted to be upstream in the MAPK pathway, whereas Erk1/2 was predicted to be the central signaling node connecting the MAPK pathway with the network of transcriptional regulators via the induction of *Olig2* expression (Fig. 3d).

To test these predictions we used the potent Pyk2 inhibitor PF-431396^28^ and the MEK-ERK inhibitor U0126 and measured *Olig2* expression. Exposure of NSCs to Chi3l3 in the presence of PF-431396 or U0126 resulted in significant inhibition of Chi3l3-induced *Olig2* expression (Fig. 3e, g). In line with our results from pharmacological inhibition, knockdown of *Ptk2b* (Supplementary Figure 6A) and overexpression of dominant-negative Erk2-K54R (Supplementary Figure 6B) resulted in significantly decreased Chi3l3-induced *Olig2* expression (Fig. 3f, h). Furthermore, both, pharmacological inhibition of Pyk2 signaling and knockdown of *Ptk2b* expression in NSCs, resulted in significant reduction of Chi3l3-induced oligodendrogenesis (Fig. 3i, j), linking the Chi3l3-dependent activation of the MAPK pathway with its downstream transcriptional targets and function in oligodendrogenesis.

### Chi3l3 activates EGFR to promote oligodendrogenesis

**Fig. 4:**
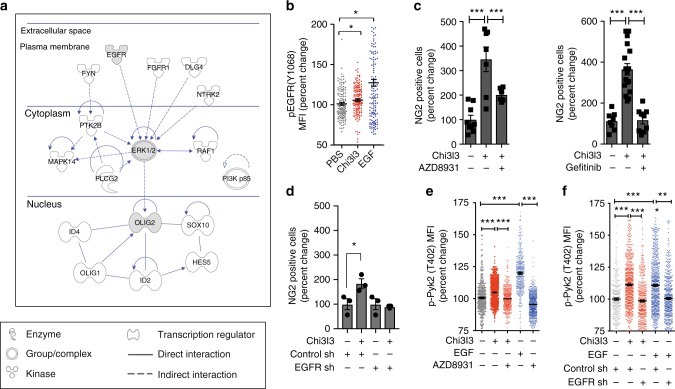
Chi3l3 promotes oligodendrogenesis by inducing EGFR signaling. **a** ingenuity pathway analysis showing membrane-bound receptors (EGFR, FGFR1, DLG4 (PSD95), NTRK2, FYN) upstream of Chi3l3-induced MAPK signaling (Ptk2b (Pyk2), MAPK14 (p38MAPK), PLCG2 (PLCγ2), RAF1 (c-Raf), ERK1/2, PI3K p85) and pro-oligodendrogenic transcriptional pattern (*Olig1, Olig2, Sox10, Hes5*, *Id2*, *Id4*). **b** Mean fluorescence intensity (MFI) quantification of individual neural stem cells (NSCs), activated by Chi3l3, EGF, or PBS (control) for 15 min, and immunostaining for phospho (p)-specific EGFR (Tyr1068). One-way-ANOVA with Dunn’s multiple comparison test; **c** Quantification of NSC-derived NG2^+^ oligodendrocyte precursor cells after 3 days of differentiation in the presence of Chi3l3 ( + ) or PBS (−) and EGFR kinase inhibitor AZD8931 ( + , left) and Gefitinib ( + , right) or DMSO (−). One-way ANOVA with Bonferroni’s multiple comparison test; **d** Quantification of NG2^+^ oligodendrocyte precursor cells derived from differentiating NSCs, transfected with control shLVPs or *EGFR* shLVPs and treated with Chi3l3 ( + ) or PBS (−). Deficiency in EGFR signaling significantly reduced Chi3l3-induced oligodendrogenesis. One-way ANOVA with Bonferroni’s multiple comparison test; **e** Mean fluorescence intensity (MFI) quantification of NSCs, activated by Chi3l3 ( + ), EGF ( + , positive control) or PBS (−, negative control) in the presence of EGFR inhibitor AZD8931 for 15 min, and immunostaining for phospho (p)-specific Pyk2 (Tyr402). One-way ANOVA with Bonferroni’s (**d**, neuroblasts, red) test; **f** Mean fluorescence intensity (MFI) quantification of NSCs transfected with control shLVPs or *EGFR* shLVPs, activated by Chi3l3 ( + ), EGF ( + , positive control) or PBS (−, negative control for 15 min, and immunostained for phospho (p)-specific Pyk2 (Tyr402). Deficiency in EGFR signaling reduced Pyk2 phosphorylation in neural stem cells. All values are expressed as percent change to PBS-treated NSCs. One-way ANOVA with Bonferroni’s multiple comparison test; mean ± s.e.m; *p < 0.05; **p < 0.01; ***p < 0.005

To predict the membrane-bound activators through which Chi3l3 induced the MAPK pathway, we performed an upstream-analysis using IPA. The method identified five membrane-bound kinases that are upstream of the signaling modules activated by Chi3l3 and that could serve as potential receptors (Fig. 4a). Among the identified upstream molecules was the receptor tyrosine kinase EGFR, also known as ErbB1 or HER-1.

EGFR can homodimerize or heterodimerize with other ErbB family members upon binding of peptide ligands, triggering tyrosine phosphorylation and activation of downstream signaling cascades. EGFR regulates a broad spectrum of cellular functions including oligodendrogenesis and remyelination^29–31^. We investigated the phosphorylation status of EGFR in differentiating NSCs, and found that Chi3l3 and its canonical ligand EGF significantly induced phosphorylation of the EGFR at tyrosine 1068 after 15 min (Fig. 4b). Next, we tested whether pharmacological inhibition of EGFR signaling affected Chi3l3-mediated oligodendrogenesis in vitro. Here we used the pharmacological inhibitors AZD8931/Sapatinib and Gefitinib, blocking the complete EGFR family and EGFR, respectively. Both AZD8931 and Gefitinib resulted in inhibition of Chi3l3-induced oligodendrogenesis (Fig. 4c). Similarly, knockdown of EGFR expression in NSCs (Supplementary Figure 6C) significantly reduced Chi3l3-mediated oligodendrogenesis (Fig. 4d). Furthermore, inhibition of EGFR signaling by pharmacological inhibition or knockdown resulted in inhibition of Chi3l3- and EGF-dependent Pyk2 phosphorylation (Fig. 4e, f), indicating that Pyk2 is a downstream target of EGFR activity during Chi3l3-dependent NSC activation.

These data indicate that Chi3l3 induces oligodendrogenesis by activating Pyk2 in an EGFR-dependent manner.

### Chi3l3 infusion induces oligodendrogenesis in vivo

**Fig. 5:**
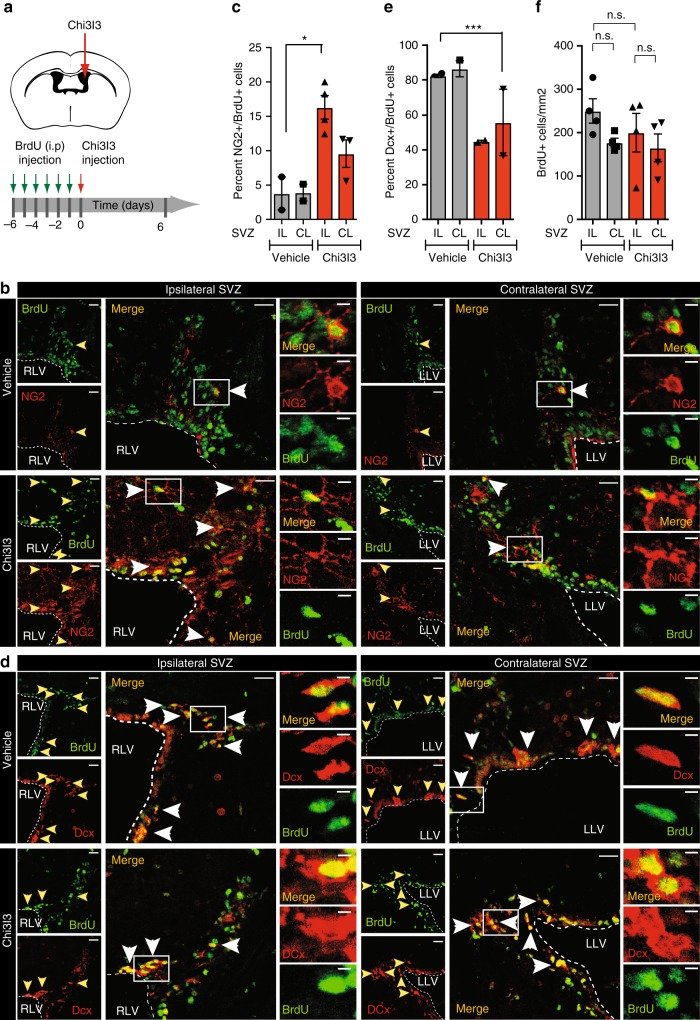
Chi3l3 infusion induces pro-oligodendrogenic fate of subventricular zone neural stem cells in vivo. **a** Protocol of BrdU labeling via intraperitoneal (i.p.) injection and Chi3l3 administration. **b**–**f** Representative confocal images **b**, **d** and quantification **c**, **e**, **f** of the ipsilateral (IL) or contralateral (CL) subventricular zone (SVZ) of naive mice infused with Chi3l3 or PBS (vehicle) and immunostained for BrdU (green) and NG2 (**b**, oligodendrocyte precursor cells, red) or Dcx (**d**, neuroblasts, red). IL SVZ of Chi3l3-treated mice were compared with IL SVZ of vehicle-treated mice as well as the CL site of the same mouse. Dashed lines mark the ventricular wall of the right or left lateral ventricle (RLV; LLV). Scale bar (left), 50 μm. Double-positive cells are indicated by yellow and white arrowheads and representative cells (box) are shown as high magnification images (right). Scale bar (right), 5 μm. Quantifications show a significantly higher percentage of BrdU/NG2 double-positive cells **c** and reduced percentage of BrdU^+^/Dcx^+^ cells **e** in the ipsilateral SVZ of Chi3l3 infused mice indicating pro-oligodendrogenic modulation of SVZ stem cell fate choice in vivo. **f** Quantification of the total number of BrdU labeled cells/mm^2^ in the SVZ showed no significant difference between Chi3l3 or vehicle infusion in the ipsilateral or contralateral SVZ. (One-way ANOVA with Turkey’s post hoc test; **c** *n* = 2 mice (Control IL, CL), *n* = 4 (Chi3l3 IL), and *n* = 3 (chi3l3 CL); **e** *n* = 2 mice per group, *n* = 4 mice per group; mean ± s.e.m. *p < 0.05; **p < 0.01; ***p < 0.005)

To test whether Chi3l3 affects NSC fate choice in vivo, we pulsed naive mice with 5-bromo-2’deoxyuridine (BrdU) for 6 days to label SVZ-NSCs and then followed differentiation into their cell fates after stereotactical injection of recombinant Chi3l3 (500 ng/mouse) or vehicle into the right lateral ventricle (RLV) (Fig. 5a). The percentage of NSCs that differentiated into OPCs was quantified for the ipsilateral (IL) and contralateral (CL) SVZ of Chi3l3 and vehicle-infused mice^18,32–34^. The IL SVZ of Chi3l3-injected mice was compared with the IL SVZ of vehicle-treated mice as well as the CL SVZ of the same mouse. We found that the percentage of newly formed NG2^+^ BrdU^+^ OPCs was significantly increased (Fig. 5b, c), whereas the percentage of NSC-derived Dcx^+^ BrdU^+^ neuroblasts was significantly decreased (Fig. 5d, e) in the ipsilateral but not contralateral SVZ when compared with vehicle-infused mice. The overall number of BrdU^+^ cells in the ipsilateral and contralateral SVZ of Chi3l3- and vehicle-treated mice was not significantly altered (Fig. 5f). We confirmed that Chi3l3 had no effect on NSC proliferation in vivo by injecting recombinant Chi3l3 (500 ng/mouse) or vehicle into the RLV of mice. Proliferating NSCs were labeled by pulsing mice with BrdU 12 and 24 h after Chi3l3 infusion. Mice were killed 24 h after the last pulse and the number of BrdU-incorporating cells in the SVZ of both groups were quantified. In line with our results in vitro, Chi3l3 did not significantly affect proliferation of SVZ cells in vivo (Supplementary Figure 7).

Thus, infusion of Chi3l3 into the SVZ of naive mice induces fate choice of NSCs towards OPCs at the expense of neuroblasts and without affecting NSC proliferation in vivo.

### Chi3l3 affects oligodendrogenesis and severity of EAE

**Fig. 6:**
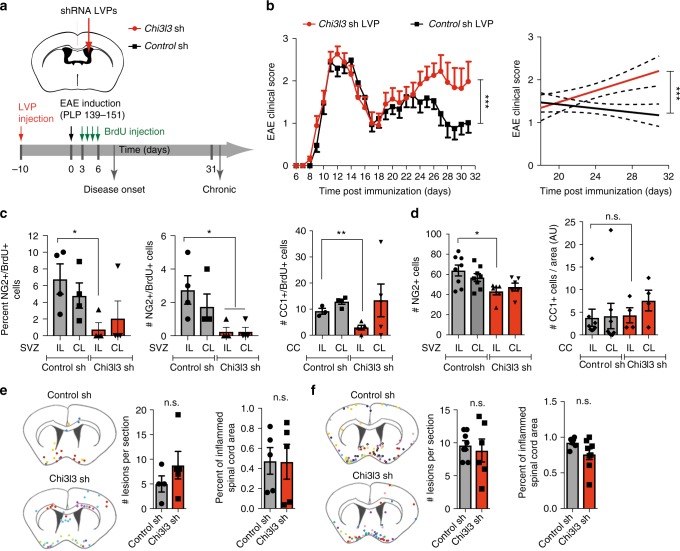
Subventricular zone-derived Chi3l3 affects EAE severity and oligodendrogenesis. **a** Protocol of shRNA lentiviral particle (LVP) administration, BrdU injection, and EAE induction. **b** Clinical score (left) and linear regression curves (right) of mice treated with control shRNA LVPs (black line) or Chi3l3 shRNA LVPs (red line) and immunized with PLP_139–151_. Dashed lines indicate 95% confidence intervals. (*n* = 23 control shRNA LVP and 27 Chi3l3 shRNA LVP mice per group; two-way ANOVA; **c** Quantifications of percentage (left) and number (right) of NG2^+^/BrdU^+^ double-positive cells in the ipsilateral (IL) and contralateral (CL) SVZ of Chi3l3 shLVP and control shLVP mice killed at onset of disease in vivo. (*n* = 4 per group; two-tailed Student’s *t* test; **d** Quantifications of and number of NG2^+^ cells in the ipsilateral (IL) and contralateral (CL) SVZ of Chi3l3 shLVP and control shLVP mice sacrificed during chronic EAE (*n* = 8 control shRNA mice, *n* = 6 Chi3l3 shRNA mice; two-tailed Student’s *t* test. **e**, **f** Localization (left) and quantification of inflammatory lesions in brain sections (middle) and spinal cord sections (right) of Chi3l3 shLVP and control shLVP mice killed at EAE onset (**e**; *n* = 4 mice control shRNA, *n* = 5 mice Chi3l3 shRNA (left, brain), *n* = 5 mice per group (right, spinal cord; two-tailed Student’s t-test; mean ± s.e.m) and during chronic EAE (**f**, *n* = 7 mice control shRNA mice, *n* = 8 Chi3l3 shRNA mice (left, brain) and *n* = 8 mice control shRNA mice, *n* = 6 Chi3l3 shRNA mice (right, spinal cord); two-tailed Student’s *t* test). Schematic diagram showing localization of inflammatory lesions of individual mice, each color represents an individual mouse. (mean ± s.e.m.; *p < 0.05; **p < 0.01; ***p < 0.005)

To investigate the role of SVZ-derived Chi3l3 during autoimmune demyelination, we used lentivirus-delivered small hairpin RNAs (shRNA LVPs) to silence *Chi3l3* expression inside the SVZ prior to induction of relapsing-remitting EAE (Chi3l3-sh mice, control-sh mice). Mice were pulsed with BrdU on days 3–6 after immunization and then killed at disease onset, or on day 32 when they had entered the chronic EAE phase. Histopathological analysis was performed on CNS tissue to assess the degree of oligodendrogenesis, myelination, and lesion load (Fig. 6a).

Control-sh mice developed acute disease peaking at 12–14 dpi followed by initial recovery, relapse, and subsequent remission at dpi 26, as expected in this model^12,18^. In contrast, Chi3l3-sh mice developed EAE earlier than control mice, followed by a similar disease course during the acute EAE phase, but significantly increased disease severity during the chronic phase (Fig. 6b).

During disease onset, the percentage and absolute numbers of newly formed NG2^+^BrdU^+^ OPCs as well as newly formed CC1^+^BrdU^+^ mature oligodendrocytes in the ipsilateral periventricular area were significantly decreased in Chi3l3-sh mice compared with control-sh mice (Fig. 6c). During chronic EAE Chi3l3-sh mice showed decreased numbers of NG2^+^ cells in the periventricular area (Fig. 6d), whereas the number of newly formed CC1^+^ BrdU^+^ mature oligodendrocytes and Luxol Fast Blue myelin staining (Supplementary Figure 8) was unchanged.

The number of inflammatory lesions was not significantly altered in sections of brain and spinal cord tissue at onset or chronic disease (Fig. 6e, f). Furthermore, we did not observe activation of the central (Supplementary Figure 9A, Supplementary Figure 10A) or peripheral immune system (Supplementary Figure 9B, Supplementary Figure 10B). Thus, the observed decrease in OPC generation in Chi3l3-sh mice is consistent with a mechanism of Chi3l3-induced clinical recovery that involves oligodendrogenesis, but not alterations of the immune system.

### Human CHIT1 and CHI3L1 induce oligodendrogenesis in vitro

**Fig. 7:**
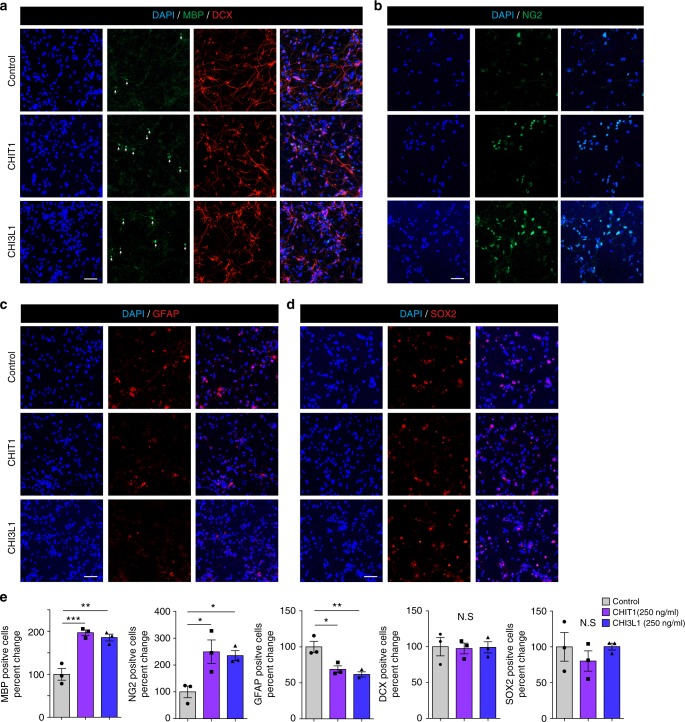
Human CLPs directly promote oligodendrogenesis. Human neural stem cells (NSCs) were cultured in the presence of PBS (control), human CHI3L1 and human CHIT1 at 250 ng/ml for 14 days. **a**–**c** Cells were immunostained for MBP (**a**, oligodendrocytes, green), DCX (**a**, neurons, red), NG2 (**b**, OPC, green), GFAP (**c**, astrocyte, red), SOX2 (**d**, NSC, red) and nuclear stain DAPI (blue). Scale bar, 50 μm. **e** Quantification of MBP^+^, NG2^+^, GFAP^+^, DCX^+^, and SOX2^+^ NSCs after exposure to human Chi3L1or human Chit1. Exposure of differentiating NSCs to CHI3L1 and CHIT1 led to significant increase in oligodendrogenesis. Values are expressed as percent change to PBS-treated NSCs (data are representative of three independent experiments. one-way ANOVA with Dunnett’s multiple comparison test; mean ± s.e.m *p < 0.05; **p < 0.01; ***p < 0.005; N.S. = not significant

To examine the potential clinical relevance of our findings, we analyzed the effects of the human paralogues, CHI3L1 and CHIT1, on differentiation of human NSCs in vitro. Like murine Chi3l3, both human paralogues significantly induced differentiation of human NSCs into NG2^+^ OPCs and MBP^+^ mature oligodendrocytes (Fig. 7a, b, e) and reduced astrogliosis (Fig. 7b, e). The percentage of DCX^+^ and SOX2^+^ cells was unchanged (Fig. 7a, d, e). These results show that human paralogues to murine Chi3l3 are also capable of inducing oligodendrogenesis.

## Discussion

**Fig. 8:**
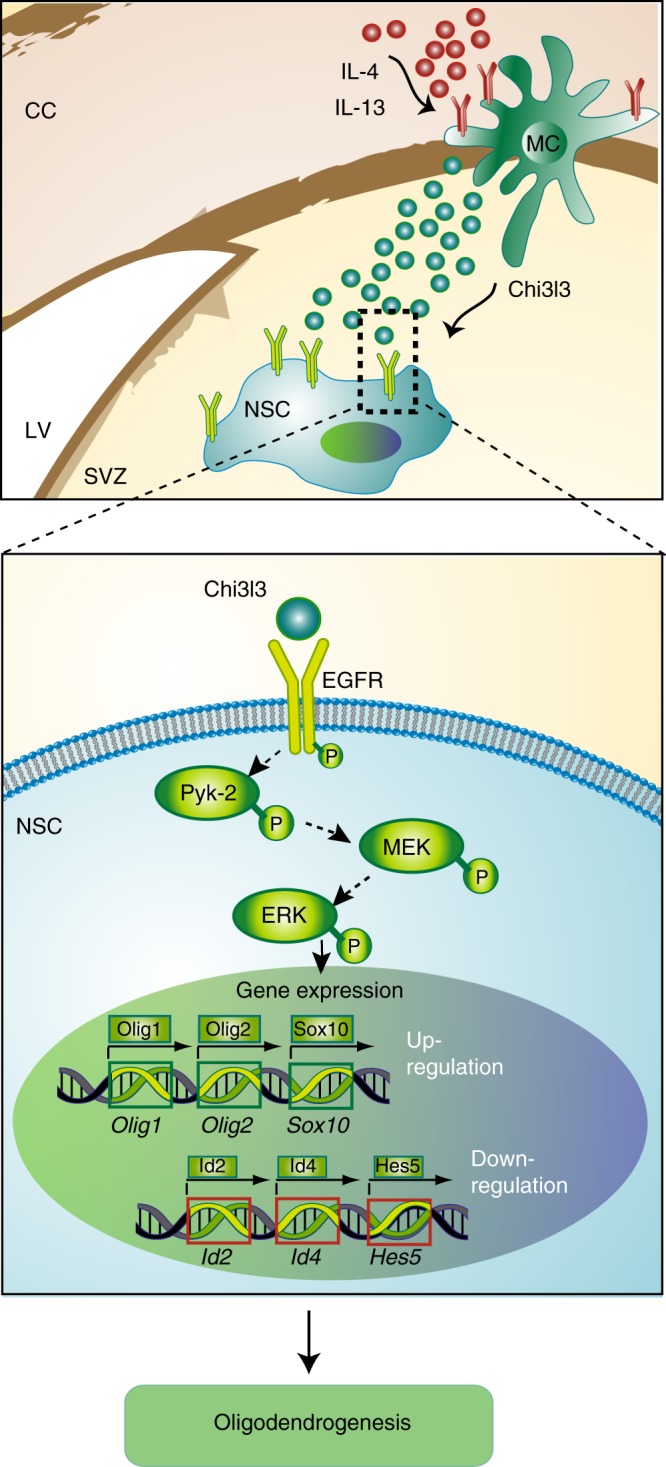
Proposed model of Chi3l3-induced oligodendrogenesis. In the central nervous system, Chi3l3 is expressed and secreted by alternatively activated myeloid cells (MC), including resident microglia and infiltrating macrophages. These cells are located in the corpus callosum (CC) and in close proximity to the subventricular zone (SVZ) of the lateral ventricle (LV). Chi3l3 binds to epidermal growth factor receptor (EGFR) on the surface of endogenous neural stem cells (NSCs) and activates MEK/ERK signaling and a pro-oligodendrogenic transcription factor pattern in a Pyk2-dependent manner, leading to oligodendrogenesis

Our data suggest that the dynamics of Chi3l3 expression controls oligodendrogenesis and affects disease severity throughout the course of autoimmune demyelinating disease. Mechanistically, our results provide evidence that Chi3l3, a mammalian lectin previously known only as a marker for M2 microglia/macrophages, is an activator of EGFR and induces oligodendrogenesis by activating MAPK signaling and pro-oligodendrogenic transcriptional regulation in a Pyk2-dependent manner (for the presumptive mechanistic pathway, see Fig. 8).

The regulation of NSC fate choice largely depends on the SVZ microenvironment^3,8,9^, which is significantly shaped by soluble mediators present in the cerebrospinal fluid (CSF)^35^ or produced by local microglia^36^. Both local microglia and the CSF composition undergo dynamic changes in response to different physiological and pathophysiological states^37^. However, little is known about how these changes in the local microenvironment affect neural stem cell behavior. Here, we show that endogenous Chi3l3 is expressed by CD11b^+^ myeloid cells in the SVZ and its expression parallels the dynamic regulation of oligodendrogenesis during the course of autoimmune demyelinating disease^38^, reaching its highest expression at peak disease and decreasing thereafter.

Our work shows that infusion of Chi3l3 into the CSF of healthy mice with low Chi3l3 expression resulted in a modulation of NSC fate choice toward the oligodendrocyte lineage without affecting proliferation in vivo. In line with these results, and strongly supporting a role for Chi3l3 as an essential regulator of oligodendrogenesis in vivo, local suppression of EAE-induced Chi3l3 expression in the SVZ led to decreased oligodendrogenesis. It is likely that shRNA LVPs integrated into microglia lining the ventricular wall, in line with earlier studies using a similar experimental design^39,40^. These presumptive target cells are known to highly express Chi3l3. However, further studies are needed to provide direct evidence for the exact localizations of virus infection and therefore, of Chi3l3 knockdown.

In accordance to our in vitro data, suppression of *Chi3l3* expression blocked differentiation of NSCs into OPCs and further into mature oligodendrocytes during onset of clinical signs in EAE mice. However, the fact that the OPC differentiation as well as differences in normal myelin staining were not observed during chronic EAE may indicate that OPC differentiation and remyelination are either overcome or inhibited by other factors of the chronic microenvironment. We must bear in mind that although the in vitro data strongly support the notion that Chi3l3 acts directly on NSC, our in vivo data do not exclude potential indirect effects of the Chi3l3 on oligodendrogenesis. EGFR is expressed on choroid plexus epithelial cells, ependymal cells and type-C cells of the SVZ^41^. Thus, in the in vivo context, Chi3l3 may also act by targeting those cells that are directly in contact with the protein.

*Chi3l3* knockdown increased EAE disease severity during the onset and chronic phase of the disease without altering activation of the peripheral and central immune system. However, a surprising finding was that local knockdown of endogenous Chi3l3 in the SVZ did not affect severity or duration of the acute phase, although Chi3l3 expression was maximal during acute EAE. A potential explanation is that Chi3l3 is also expressed by peripheral monocytes and macrophages^42^. Thus, a local decrease of Chi3l3 expression could potentially be compensated by the influx of Chi3l3-expressing peripheral monocytes during acute EAE. As these cells die rapidly^43^, the local decrease of Chi3l3 may re-emerge during chronic EAE and may then result in alterations in the clinical score. In addition, the clinical consequences of Chi3l3-induced oligodendrogenesis could be either contemporaneous, or results from knockdown in an earlier disease phase. It is a limitation of the present study that we cannot distinguish all cell-specific and disease phase-specific effects of Chi3l3 expression in the CNS. Further limitations include the fact that although in this model, clinical severity correlates with the magnitude of brain lesions^44^, the EAE score largely reflects inflammation and tissue damage in the lumbar spinal cord. Thus, further studies investigating Chi3l3-mediated changes in the spinal cord of EAE mice are ongoing to fully explain the causality between Chi3l3-mediated effects and the EAE clinical score. In addition, the cuprizone model of toxic demyelination will be useful to test specific mechanistic aspects of Chi3l3-mediated oligodendrogenesis in the periventricular area and the corpus callosum. It hast to be mentioned, that microglial Chi3l3 expression highly increases during EAE but remains unchanged during the course of cuprizone-mediated demyelination and subsequent remyelination^45^ pointing towards the importance of the autoimmune activation cascade in initiating the Chi3l3-mediated repair processes.

Our in vitro data show that Chi3l3 induces oligodendrogenesis by directly targeting pro-oligodendrogenic NSC differentiation without affecting proliferation, self-renewal capacity or cell death. This effect was promoted by activating pro-oligodendrogenic and pro-myelination transcription factors such as *Olig1*, *Olig2*, and *Sox10* and by diminishing expression levels of negative regulators of oligodendrogenesis such as *Id2*, *Id4* and *Hes5*. Chi3l3-mediated pro-oligodendrogenic fate choice by activating the MAPK pathway of cell growth and differentiation by means of phosphorylation of signaling nodes including Pyk2 (Tyr^402^) and Erk1/2 (Thr^202^/Tyr^204^).

Pyk2 is a tyrosine kinase of the MAPK pathway^46^ and a signaling molecule with known functions in tissue regeneration and cancer^28,47^, however its function in NSCs had been unknown to date. Strikingly, our phosphor-protein measurements and inhibition studies demonstrate that Pyk2 signaling is required for Chi3l3-induced *Olig2* expression and abrogated Chi3l3-dependent oligodendrogenesis suggesting that Pyk2 has an important role in Chi3l3-induced modulation of NSC fate choice. Whether Pyk2 signaling is also central to NSC functions induced by stimulants other than Chi3l3 has to be elucidated.

Apart from differentiation, activation of the endogenous NSC compartment also leads to proliferation, migration, and survival of NSCs. Signaling by the EGFR, which is expressed on the cell membrane of developing and adult NSCs, regulates all of these effects^29,48^. Several different ligands of EGFR including EGF, heparin-binding EGF-like growth factor (HB-EGF) and TGFα^48^, have been identified, each controlling a specific function by signaling via a particular downstream pathway. HB-EGF and TGFα are associated with proliferation and survival of progenitors^49^. In contrast, activation of EGFR signaling by EGF induces oligodendrogenesis^50^ and was thought to be a promising agent to enhance white matter repair. Counterintuitively, antibody-mediated blockade of EGF in vivo was shown to ameliorate EAE and induce oligodendrogenesis^51^. Furthermore treatment with EGF induced substantial proliferation and migration of progenitors, which led to diffuse hyperplasia of glial progenitors in the white matter without differentiation into mature oligodendrocytes^50,52^.

Our data suggest that Chi3l3, acting as a regulator of EGFR, may have a better therapeutic potential, because it induces oligodendrogenesis without affecting proliferation, survival, and self-renewal. Our phospho-protein analysis revealed EGFR as an essential activator for Chi3l3-mediated oligodendrogenesis in NSCs, and links Chi3l3-mediated EGFR activation with the induction of main MAPK and PI3K signal transducers such as Erk1/2 and p85-PI3K. Our data showed that Chi3l3 leads to EGFR phosphorylation at Tyr1068, a site that is particularly important for the induction of the Ras/MAPK pathway by EGFR^48^. Importantly, blockade of EGFR activation by specific inhibitors and knockdown of EGFR in NSCs abrogated Pyk2 phosphorylation, which was the earliest event detected in our reverse-phase protein array kinetic experiment. We conclude that EGFR activation is an immediate and essential event triggered by Chi3l3, making Chi3l3 a compelling candidate as an EGFR ligand or co-ligand.

The extracellular domain of EGFR is heavily *N*-glycosylated and the glycosylation pattern is implicated in receptor dimerization and subsequent activation^53,54^. Interestingly, the crystal structure of Chi3l3 revealed a saccharide-binding site^55^ and functional studies showed that Chi3l3 exhibited binding specificity to saccharides with free amine groups, including glucosamine (GlcN) and GlcN-polymers^56^, making it compatible with binding to EGFR. However, further research is needed to elucidate whether Chi3l3 acts as a direct ligand to EGFR or activates it indirectly. Taken together, our data provide evidence that the Chi3l3-EGFR-Pyk2 axis constitutes a hitherto unknown pathway that may be activated to induce pro-oligodendrogenic fate choice without the induction of stem cell proliferation.

The family of mammalian CLPs also includes the structurally and functionally related paralogues: murine Chi3l1 and human CHI3L1 and CHIT1. Chi3l1 knockout mice have a more severe course of MOG-induced EAE with increased demyelination^36^, suggesting that several members of this protein family of CLPs may have regenerative functions.

Similar to murine Chi3l3 and Chi3l1, their human paralogues CHI3L1 and CHIT1 are highly abundant in the CNS during acute demyelinating diseases, including relapsing-remitting MS (RRMS) and neuromyelitis optica, but not chronic MS^37^. In particular, CHI3L1 levels in the CSF were found to be significantly higher in active progressive MS compared with inactive progressive MS^57,58^, correlating with clinical and radiological disease activity in RRMS patients^38,58^, and predicting conversion from clinically isolated syndrome to MS^59^. CHI3L1 protein levels in the CSF strongly correlated with markers of CNS tissue damage and tissue remodeling including osteopontin, NFL, and the myelin protein MBP^41^, whereas CHIT1 expression only correlated with CSF levels of MBP^42^. Although CHI3L1 and CHIT1 have been widely studied as biomarkers of disease progression and tissue damage in MS, their function in the CNS is not yet clear. We now show that, like murine Chi3l3, human CHI3L1 and CHIT1 induce oligodendrogenesis in human NSCs in vitro. Notably, this may suggest that the in vivo expression of CLPs, particularly during active MS participate in the disease process as an attempt to initiate repair. This intriguing association will spark new research into the biological role of these known biomarkers.

However, embryonic mouse and human NSCs differ from each other as well as from their adult counterparts^60–62^. This may constitute a potential limitation, as embryonic NSCs expresses a distinct set of biomarkers^60^ and are more plastic^63^ than adult NSCs. Thus, future studies are needed to prove that CLPs induces oligodendrogenesis by the above-described mechanism in mice in vivo and caution has to be exercised when translating the mechanistic findings of this study in a human and particularly into a disease setting. Although direct evidence for the functional role of human CLPs in the demyelinated CNS in vivo is still lacking, human CHI3L1 and CHIT1 may also participate in pro-oligodendrogenic fate choice during MS.

In summary, we have identified a hitherto unknown CNS regulatory circuit by which microglia activate oligodendrogenesis in endogenous NSCs through CLP-dependent mechanisms. Chi3l3-induced stimulation of oligodendrogenesis was mediated by induction of pro-oligodendrogenic transcription factors downstream of EGFR-Pyk2-modulated MAPK-signaling pathways and correlated with amelioration of neurological symptoms in vivo. Thus, targeting the Chi3l3-EGFR-Pyk2 signaling axis may represent a therapeutic strategy to influence fate choice in NSCs and promote oligodendrocyte repair in CNS diseases involving white matter injury.

## Methods

### Animals

Female SJL/J/6 mice were purchased from the Jackson Laboratory (Bar Harbor, ME, USA). All animals were housed in pathogen-free facilities. All experiments were performed with the approval of the local animal welfare committees (Harvard Medical Area Standing Committee on Animals, and LAGeSo, Berlin) and in accordance with national and international guidelines to minimize discomfort to animals (NIH and 86/609/EEC).

### Cell lines

293 T cells were purchased from Life Technologies. These cells were authenticated by Life Technologies. H9 hESC-derived NSCs (H9, N7800–100) were purchased from Thermofisher scientific. These cells were authenticated by the vendor and additional human genome sequencing and microarray analysis was performed (data not shown). BV-2 cells were acquired as a kind gift from F. Aloisi (Instituto Superiore di Sanità, 00161 Rome, Italy. These cells were positively tested for microglial markers, however no further authentication was performed. Testing for mycoplasma contamination was not performed.

### Generation of lentiviral particles

Chi3l3 Mission® shRNA plasmid DNA in a pLKO.1 vector backbone (clone ID: NM_009892.1–567s1c1), Ptk2b Mission® shRNA plasmid DNA in a pLKO.1 vector backbone (clone ID: TRCN0000023632), EGFR Mission® shRNA plasmid DNA in a pLKO.1 vector backbone (clone ID: SHCLNG-NM_207655), and pLKO.1-puro control DNA were purchased from Sigma. A dominant-negative Erk2^64^ (dnErk2_K54R) was cloned into a lentiviral gateway (Invitrogen) destination vector that contains the sequence of GFP in the N-terminal position. The resulting expression plasmid contains a fusion of dnErk2_K54R and GFP. The same expression plasmid backbone was used for the control, from which only GFP is expressed. Cells expressing GFP- dnErk2_K54R in addition to endogenous Erk1/2, show reduced phosphorylation of Erk1/2 targets (data not shown). Lentiviral particles pseudotyped by the vesicular stomatitis viral envelope were generated by transfecting 293 T cells (Life Technologies). Supernatants were collected after 48 h, filtered through a 0.45 μm polyvinylidene difluoride filter and concentrated overnight. The viral titer was determined according to the manufacturer’s instructions, and stored at −80 °C.

### Stereotactic injection of Chi3l3 or lentiviral particles

Recipient mice (5–7 weeks old) were anesthetized with ketamine (200 mg per kg) and xylazine (10 mg per kg). Heads were secured in a stereotaxic head frame (Stoelting). A small hole was drilled into the mouse skull, meninges were locally removed with H_2_O_2_, and a 10 μl Hamilton syringe with a 29 G needle was inserted into the right lateral ventricle. Recombinant Chi3l3 (100 ng in 5 μl phosphate-buffered saline; PBS, RnD Systems) or PBS (5 μl) or lentiviral particles (1 × 10^7^ IU; shChi3l3 or shControl (nontargeting) virus) were injected at a flow rate of 1 μl per min at the following coordinates: anteroposterior, 0.34 mm; lateral, 1.2 mm; and dorsoventral, 2.4 mm. After completion of injection, the needle was left in place for an additional 5 min and then withdrawn at a rate of 0.5 mm per minute to prevent leakage. The resulting wound was sutured with surgical nylon, and mice were inspected daily as part of the postoperational care.

### EAE induction

Mice were immunized subcutaneously in two sites (left and right flanks) with 150 μg of PLP 139–151 (New England Peptide, Gardner, MA) peptide that was emulsified in complete Freund’s adjuvant (CFA; Sigma-Aldrich) containing 200 μg Mycobacterium tuberculosis (Difco), followed by 200 ng pertussis toxin (PT; List Biological Laboratories) in 0.2 ml PBS by intraperitoneal (i.p.) injections at the time of immunization and 48 h later. Control mice were immunized with CFA, followed by PT injection. Mice were scored daily by an independent blinded researcher as follows: 0, no clinical signs; 0.5, partial loss of tail tone; 1, loss of tail tone; 1.5, poor righting ability; 2, hind-limb weakness; 3, hind-limb paralysis; 4, quadriparesis; and 5, moribund. Mice found dead were assigned a score 5 each day until the end of the experiment.

### In vivo analysis of NSC differentiation and proliferation

For determination oligodendrogenesis in vivo naive SJL mice were injected i.p. with BrdU (120 mg per kg body weight per day, Sigma Aldrich)^12,18^. In brief, mice were pulsed for 6 consecutive days and subjected to stereotactic injection of recombinant Chi3l3 1 day after the last BrdU injection as described below. Mice were killed 6 days after the stereotactic injection and CNS tissue was preserved and subjected to immunohistochemical analysis. For determination of NSC proliferation, mice were pulsed with BrdU (300 mg per kg body weight) 12 and 24 h after stereotactic injection of recombinant Chi3l3 into naive mice. Mice were killed 24 h after the last BrdU injection, CNS tissue was preserved and subjected to immunohistochemical analysis. All treatments following stereotactical injection were performed by an independent researcher in a blinded fashion.

### Immunostaining

Mice were deeply anesthetized in a CO_2_ chamber or lethally anesthetized with a mixture of ketamine (415 mg per kg; Actavis Germany) and xylazine (9.7 mg/lg; Bayer Health Care Germany) and then transcardially perfused with ice-cold PBS. Brains and spinal cord were removed and snap frozen in liquid nitrogen or fixed with 4% parafformaldehyde (PFA) and cryopreserved in sucrose. Tissues were then stored at −80 °C until further use. For immunohistochemical analysis, tissues were cut into sections of 10 or 25 μm thickness in a cryostat and subjected to further procedures. For immunocytochemical analysis, culture medium was removed from adherent cells and subjected to further procedures.

For immunostaining, cells or tissues were fixed with 4% PFA for 10 min, washed with PBS for 15 min, blocked with 8% horse serum, 3% BSA and 0.3% Triton X-100 in PBS for 1 h and then incubated with primary antibodies at the indicated concentrations at 4 °C overnight. Cells or sections were then rinsed and incubated for 1 h with the appropriate Alexa Fluor 488, −594, or −647 secondary antibodies (1:500, Molecular Probes). For nuclear staining, cells or sections were incubated with TO-PRO-3 (1:1000; Life Technologies) or DAPI (1:10000; Sigma) for 1 min, washed, and covered with coverslips. Negative control sections for each animal/cell sample were treated identically, except that the primary antibodies were omitted. Regions of interest were analyzed with a confocal microscope (LSM 510 Laser Scanning Microscope and LSM 3D analysis software, Linux, or an Operetta® High Content Screening System and Columbus Image Analysis System, Perkin Elmer).

For analysis of murine tissue and cells, primary antibodies at working concentrations were: rat anti-BrdU (1:100; Accurate Chemical, cat# YSRTMCA2060GA), rat anti-CD11b (1:50; BD Biosciences, cat# 550282), mouse anti-CD45 (1:100, BioLegend, cat# 103101), rat anti-CD4 (1:200, BD Bioscience, cat# 550278) rabbit anti Chi3l3 (1:50; Stemcell Technology, cat# 60130), rabbit anti-Doublecortin (Dcx, 1:100; Abcam, cat# ab18723), mouse anti-GFAP (1:500; BD Bioscience, cat# 610566), mouse anti-Ki76-FITC (1:100; BD Bioscience, cat# 617472), mouse anti-Map2 (1:250; Sigma-Aldrich, cat# M9942), rabbit anti-NG2 (1:100; Millipore, cat# ab5320), mouse anti-O4 (1:100; Millipore, cat# MAB345), rabbit anti-p-Pyk2 (Tyr402; 1:500, CST, cat# 3291 S), rabbit anti-p-cRaf (Ser259; 1:500, CST, cat# 9421 P), rabbit anti-Sox2 (1:500, Thermo Fisher Scientific, cat# A24339), rabbit anti-p-p38MAPK (THr180/Tyr182; 1:500, CST, cat#4511 P), rabbit anti-p-PLCγ2 (Tyr1217; 1:500, CST, cat# 3871 P), rabbit anti-p-PI(3)K (Tyr458; 1:500, CST cat# 4228 P), rabbit anti-p-Erk1/2 (Thr202/Tyr204; 1:500, CST, cat# 9101), rabbit anti-p-EGFR (Tyr1068; 1:100, CST, cat# 3777).

For the analysis of human cells, primary antibodies at working concentrations were: rat anti-MBP (1: 125, Millipore, cat# MAB386), goat anti-DCX (1:250, SantaCruz, cat# sc-8066), goat anti-SOX2 (1: 250, R&D Systems, cat# AF2018), goat anti-GFAP (1: 250, Santa Cruz, cat# sc-6170), rabbit anti-NG2 (1: 125, Millipore, cat# AB5320). Secondary antibodies at working concentrations were: Alexa Fluor 488 donkey anti-rat IgG (1:250, Thermofisher scientific, cat# A21208), Alexa Fluor 488 donkey anti-rabbit IgG (1:250, Thermofisher scientific, cat# A21206), Alexa Fluor 594 donkey anti-goat IgG (1:250, Thermofisher scientific, cat# A11058).

### Confocal and non-confocal fluorescence microscopy

For quantification of in vivo stem cell differentiation and Chi3l3 expression analysis, regions of interest around the lateral ventricle were analyzed with a LSM 510 Laser Scanning microscope and LSM 3D analysis software. The periventricular area was defined as the 200 µm adjacent to the ventricular lumen all around the lateral ventricles including the entire width of the corpus callosum directly above the lateral ventricles. Cells labeled with NG2 and/or BrdU were quantified using a × 63 water-immersion objective^18^. The region of interest around the lateral ventricle labeled with Chi3l3 and/or CD11b was imaged using × 20 objective or × 63 water-immersion objective. Chi3l3 colocalization was analyzed and displayed as ortho-view of a z-stack.

Non-confocal fluorescence analysis was performed using the Operetta® High Content Screening System with a 20xWD or × 60 high NA objective and Columbus Image Analysis System (Perkin Elmer). Mean fluorescence intensity for analysis of phospho-protein staining intensity signal was calculated by automated identification individual cells and subsequent automated mean staining intensity calculation using a Columbus Image Analysis System (Perkin Elmer). A minimum of 100 cells per condition was analyzed.

### Hematoxilin and eosin stain analysis

Inflammatory lesions in brain and spinal cord were assessed with hematoxylin and eosin staining. In the brain, lesions were defined as accumulations of at least 20 mononuclear cells as previously described by Rasmussen et al.^12^. Lesions were counted and their location mapped onto a brain-diagram. Spinal cords were cut into eight segments and sectioned in order to have eight sections representative of the entire spinal cord. The degree of inflammation was determined by counting the number of quadrants that contained inflammatory lesions for each of the eight segments^65^. Data were presented as the percentage of total quadrants that contained inflammatory infiltrates.

### Murine NSC isolation and culture

Embryonic day 14.5 (E14.5) NSCs were isolated from cerebral cortices of timed C57BL/6 mouse embryos. Neural tissue was digested with the Neural Tissue Dissociation Kit (P) (Miltenyi Biotec). The cell suspension was cultured in NSC medium containing DMEM:F12 (Sigma Aldrich), 1% N2 supplement (Life Technologies), penicillin (50/ml), streptomycin (50 mg/ml), and recombinant human fibroblast growth factor/recombinant human epidermal growth factor (FGF/EGF; 20 ng/ml each, Miltenyi Biotec). For maintenance, fresh medium was added to the culture every 2–3 days and neurospheres were dissociated and passaged before reaching a critical size with necrotic cores.

For analysis of Chi3l3-dependent proliferation, NSCs were plated at 1000 cells/ml in proliferation medium containing DMEM:F12 (Sigma Aldrich), 2% B27 supplement (Life Technologies), penicillin (50 U/ml), streptomycin (50 mg/ml) and recombinant human fibroblast growth factor/recombinant human epidermal growth factor (FGF/EGF; 20 ng/ml each, Miltenyi Biotec) in the presence of 100 ng/ml recombinant Chi3l3 or PBS. Number of neurospheres per well were analyzed after 1 week of culture.

### Generation of signaling-deficient NSCs

E14.5 NSCs were infected with lentivirus stocks by spinoculation. For this, E14.5 NSCs spheres were disaggregated into single cells, and resuspended in proliferation medium containing 8 µg/ml polybrene (Sigma). Virus stocks were added at 10 multiplicity of infection, and cell–virus suspensions were centrifuged in ultra-low-adhesion round bottom 96-well plates (Corning Costar #7007) at 800 rcf for 60 min. Cell/virus pellets were incubated at 37 °C for 3 h, before they were resuspended in 2 ml medium and transferred to a six-well dish. After 72 h, puromycin or zeocin were added at 0.2 µg/ml and 25 µg/ml, respectively, to select for transfected cells. The rate of shRNA knockdown or overexpression of GFP-constructs was analyzed by qRT-PCR of the corresponding gene or flow cytometry, respectively. The fraction of GFP-expressing cells was analyzed by flow cytometry. Cells were used for experiments within five passages post transfection.

### Murine NSC differentiation and proliferation in vitro

For analysis of Chi3l3-dependent neural stem cell differentiation, NSCs were dissociated, washed and plated at 5 × 10^5^ cells per well on glass coverslips (12 mm diameter; Electron Microscopy Sciences) precoated with laminin (10 μg/ml, Life Technologies) and cultured in NSC differentiation medium containing DMEM:F12 (Sigma Aldrich), 2% B27 supplement (Life Technologies), penicillin (50 U/ml), streptomycin (50 mg/ml) in the presence of 0–1000 ng/ml recombinant Chi3l3, (R&D Systems Inc.). For functional validation of Pyk2-, EGFR-dependent oligodendrogenesis, NSCs were plated as described above and pretreated (45 min) with different concentrations of the pharmacological inhibitor (Pyk2 inhibitor PF-431396, 1 μg/ml Sigma Aldrich; EGFR inhibitor AZD9831, 200 nm, Biozol; Gefitinib, 1 μm, AstraZeneca; U0126, 10 μm, (Sigma Aldrich) or heparin (1 μg/ml, Sigma Aldrich) followed by addition of 100–500 ng/ml Chi3l3, 100–500 ng/mg hChi3L1, 100–500 ng/mg hChit1, 100 ng/ml EGF or PBS to the culture.

For analysis of cell death during Chi3l3-dependent neural stem cell differentiation, single cells were seeded as described above. PI (5 μg/ml; Roth) was added to the culture 10 min prior termination of the culture at 3 or 5 days. Cells were immediately fixed and subjected to further immunocytochemical analysis. For analysis of proliferation during Chi3l3-dependent neural stem cell differentiation, cells were pulsed with BrdU (10 μm; Sigma Aldrich) prior termination of the culture at 3 or 5 days of differentiation.

### Human neural stem cell differentiation

For analysis of hCHI3L1- or hCHIT1-dependent human neural stem cell differentiation, human NSCs (H9, N7800–100, Thermofisher scientific) were dissociated, washed and plated at 5 × 10^5^ cells on glass coverslips (diameter: 18 mm, Thermofisher scientific) precoated with poly-l-lysine (0.01% solution, Sigma) and cultured for 1 or 2 weeks in NSC differentiation medium containing NeuroBasal media, 2% B27 supplement, Glutamax, and antibiotic–antimycotic (Thermofisher scientific) in the presence of 250 ng/ml recombinant human CHI3L1 and CHIT1 (all R&D Systems Inc.).

### qRT-PCR

For quantitative real-time reverse transcription (qRT) PCR, cells or tissues were lysed, and RNA was isolated using Trizol reagent (Life Technologies) according to the manufacturer's manual. Total RNA samples were subjected to complementary DNA (cDNA) generation using the Applied Biosystems high capacity cDNA Reverse Transcription Kit. Samples were then subjected to real-time PCR analysis on an ABI 7500 System (Applied Biosystems). Genes analyzed were detected using commercially available assays from Applied Biosystems: *Ccnd1* (Mm0043258_g1), *Ccnd2* (Mm00438070_m1), *Chi3l3* (Mm00657889_mH), *Cspg4* (Mm00507256_m1), *Gapdh* (Mm00484668_m1), *Hes5* (Mm00439311_g1), *Hprt* (Mm03024075_m1), *Id2* (Mm01293217_g1), *Id4* (Mm00499701_m1), *Map2* (Mm00485230_m1), *Olig1* (Mm00497537_s1), *Olig2* (Mm01210556_m1), *Ptk2b* (Mm00552827_m1), *Sox10* (Mm01300162_m1) or primers and probes from Eurofin MWG Operon for 18 s (forward: TTCGAACGTCTGCCCTATCAA; reverse: TCCCCGTCACCCATGGT; probe: CGATGGTAGTCGCCGTGCCTACCA) and GFAP (forward: TTTCTCCAACCTCCAGATCC; reverse: CCGCATCTCCACAGTCTTTA; probe: FAM-CCAGCCTGGACACCAAATCCG-TAM). Relative mRNA level was normalized against the housekeeping genes *Gapdh*, *18* *s* or *Hprt*.

### Statistical analysis

Samples size for each experiment was chosen based on previous experiments. Samples were allocated into experimental groups at the start of each individual experiment. The in vitro experiments were not randomized. For in vivo experiments, mice were randomly subjected to each group. No data were excluded. The investigators were blinded as to experimental groups during data collection and analysis. Specifically, for in vivo experiments, EAE scoring, and subsequent tissue analysis was performed by an independent researcher, who was blinded to experimental treatment groups.

Prism software (Graph Pad Software) was used for statistical analysis. All error bars shown represent SEM. The following number of replicates were used: For Fig. 3 data are representative of three independent experiments with the following number of replicates: (A,B) *n* = 2; (C) pPyk 5 min *n* = 80 (Control) and *n* = 150 (Chi3l3), 15 min *n* = 100 (Control) and *n* = 103 (Chi3l3), 30 min *n* = 43 (Control) and *n* = 69 (Chi3l3); p-cRaf 5 min *n* = 100 (Control, Chi3l3), 15 min *n* = 99 (Control) and *n* = 101 (Chi3l3), 30 min *n* = 97 (Control) and *n* = 100 (Chi3l3); p38MAPK 5 min *n* = 72 (Control) and *n* = 105 (Chi3l3), 15 min *n* = 103 (Control) and *n* = 102 (Chi3l3), 30 min *n* = 78 (Control) and *n* = 98 (Chi3l3); p-PLCγ2 5 min *n* = 99 (Control) and *n* = 100 (Chi3l3), 15 min *n* = 102 (Control) and *n* = 101 (Chi3l3), 30 min *n* = 99 (Control) and *n* = 100 (Chi3l3); p-PI(3)K 5 min *n* = 99 (Control) and *n* = 104 (Chi3l3), 15 min *n* = 101 (Control) and *n* = 105 (Chi3l3), 30 min *n* = 88 (Control) and *n* = 47 (Chi3l3); pERK1/2 5 min *n* = 101 (Control) and *n* = 119 (Chi3l3), 15 min *n* = 102 (Control) and *n* = 107 (Chi3l3), 30 min *n* = 100 (Control) and *n* = 118 (Chi3l3); (E) *n* = 3 replicates; (F) *n* = 2 replicates (−) and *n* = 3 replicates ( + ); (G) *n* = 3 replicates; (I) *n* = 18 (−/−), *n* = 8 (−/ + ) replicates), *n* = 12 ( + /−; + / + ); (J) *n* = 3 replicates; (H) data are representative of two independent experiments with *n* = 3 replicates); For Fig. 4 data are representative of three independent experiments with the following number of replicates: (B) *n* = 93 (PBS), *n* = 98 (Chi3l3) and *n* = 108 (EGF); (C left) *n* = 9 (−/−), *n* = 7 ( + /−), and *n* = 6 ( + / + ); (C right) *n* = 8 (−/−), *n* = 17 (+ /−), and *n* = 9 ( + / + ); (D) *n* = 3 (−/ + /−), *n* = 3 ( + / + /−), *n* = 3 (−/−/ + ), and *n* = 3 ( + /−/ + ); (E) *n* = 402 (−/−/−), *n* = 1006 ( + /−/−), *n* = 648 (+ /−/ + ), *n* = 421 (−/ + /−) and *n* = 1324 (−/ + /−) individual cells; (F) *n* = 302 (−/−/ + /−), *n* = 704 ( + /−/ + /−), *n* = 450 ( + /−/−/ + ), *n* = 637 (−/ + / + /−) and *n* = 497 (−/ + /−/ + ) individual cells; In Fig. 7b linear regression analysis was performed to determine whether the differences between the groups are significant. Significant differences were assumed at the 5% level and presented as *p* values (*p* < 0.05).

### Reporting summary

Further information on experimental design is available in the Nature Research Reporting Summary linked to this Article.

## Supplementary Information

Supplementary Information

Reporting Summary

## Data Availability

The data that support the findings of this study are available from the corresponding author upon reasonable request. A Reporting Summary for this Article is available as a Supplementary Information file.
