# Supplementary material for: Chi3l3 induces oligodendrogenesis in an experimental model of autoimmune neuroinflammation
Source: Nat Commun. 2019 Jan 15;10:217. doi: 10.1038/s41467-018-08140-7 (PMC6333780; doi:10.1038/s41467-018-08140-7)
Supplement: Supplementary file 1 — Supplementary Information [file 41467_2018_8140_MOESM1_ESM.pdf]

SUPPLEMENTARY INFORMATION

Chi3l3 induces oligodendrogenesis and reduces disease severity in an experimental model of autoimmune neuroinflammation

Starossom et al.

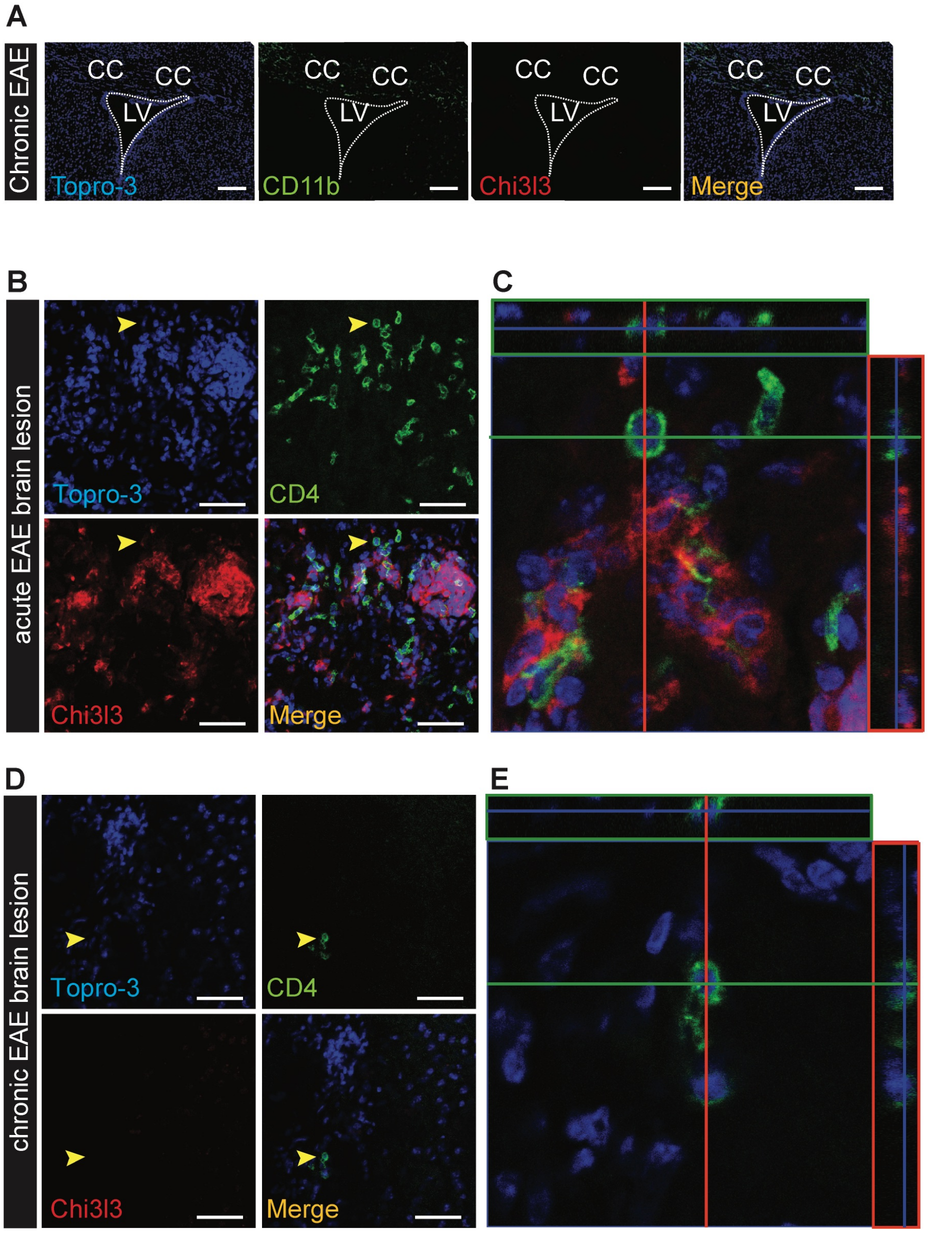

Supplementary Fig. 1. Chi3l3 is not expressed in chronic EAE and CD4^{+} T cells in periventricular lesions during EAE.

Confocal images of the Corpus callosum (CC) and subventricular zone (SVZ) of representative chronic EAE mice (**A**) and periventricular lesions of acute (12-14 dpi, **B, C**) and chronic EAE (35-40 dpi, **D, E**) mice. Dashed lines mark the wall of the lateral ventricle (LV). Sections were immunostained for the nuclear marker TO-PRO-3 (blue), Chi3l3 (red) and the T cell marker CD4 (green). (**B**) Overview- and (**C**) Ortho-view images of representative CD4-positive T cells during acute EAE. (**D**) Overview- and (**E**) Ortho-view images of representative CD4-positive T-cells during chronic EAE. Chi3l3 expression in the SVZ was only detected during acute EAE and did not co-localize to CD4 positive T cells (yellow arrowheads). Scale bar, 200 µm

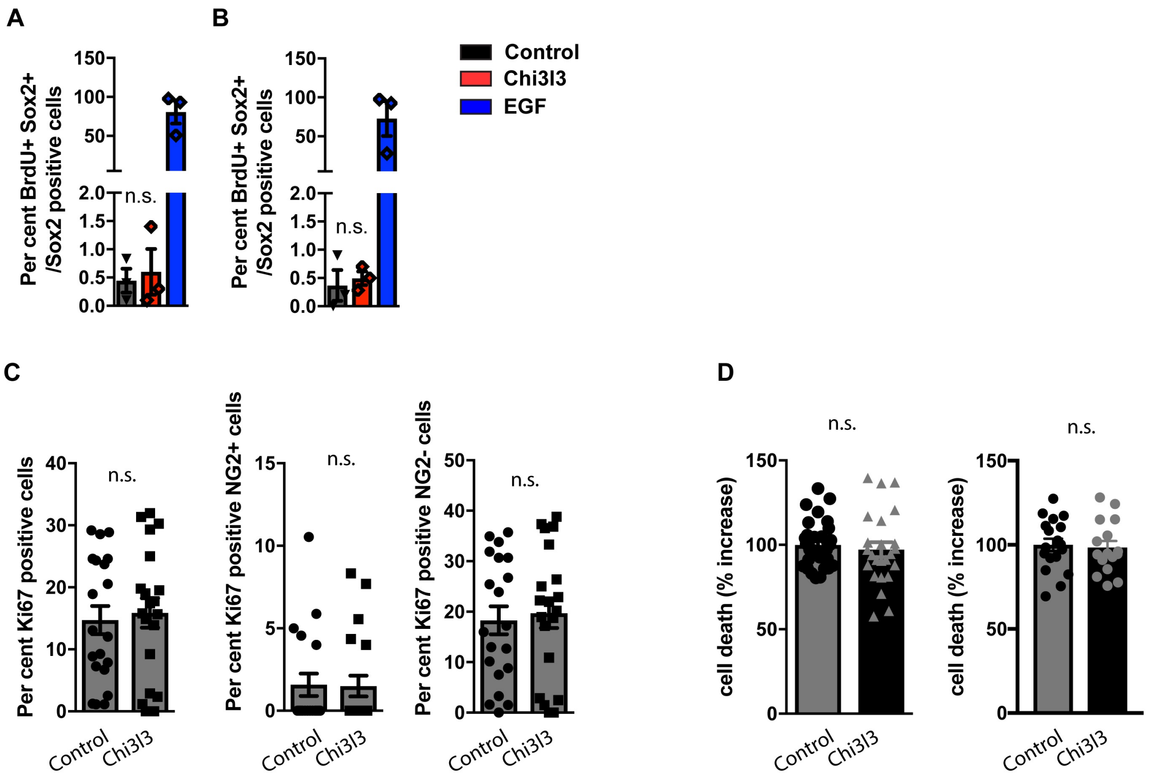

Supplementary Fig. 2. Chi3l3 does not affect proliferation and cell death of neural stem cells *in vitro.* (**A, B**) Quantification of NCSs differentiated in the presence of Chi3l3 or epidermal growth factor (EGF) for 3 days (**A**) or five days (**B**) and immunostained for the neural stem cell markers Sox2, the proliferation marker BrdU and the nuclear marker DAPI. Quantifications are displayed as percentage of BrdU^{+}/Sox2^{+} double-positive cells of total Sox2 positive cells. Exposure to Chi3l3 during NSC differentiation did not lead to significant changes in the percentage of proliferating neural stem cells while EGF induced proliferation in nearly 100% of Sox^{+} NSCs. (**C**) Quantification of NCSs differentiated in the presence of Chi3l3 for 3 days and immunostained for the progenitor lineage marker NG2 (OPCs), the proliferation marker Ki67 and the nuclear marker DAPI. Quantification is displayed as percentage of marker positive cells of DAPI positive cells. Exposure to Chi3l3 during NSC differentiation did not lead to significant changes in the percentage of total cells (left), proliferating NG2^{+} OPCs (middle) or proliferating NG2 negative cells. (**D**) Quantification of cell death in the presence of Chi3l3 for 3 days (left) and 5 days (right) and immunostained for the cell death marker Propidium Iodide and the nuclear marker DAPI. (two-tailed Student's t-test; mean ± s.e.m; Data are representative of 3 (A, B, D) and 5 (C) independent experiments with the following number of replicates: (A, B) $n = 3$; (C, % Ki67+, % Ki67+ NG2-) $n = 20$ per group; (C, % Ki67+ NG2+) $n = 19$ (Control) and $n = 20$ (Chi3l3); (D left) $n = 32$ (Control) and $n = 24$ (Chi3l3); (D right) $n = 18$ (Control) and $n = 16$ (Chi3l3); mean ± s.e.m $^{*}p < 0.05$; $^{**}p < 0.01$; $^{***}p < 0.005$; n.s. = not significant.

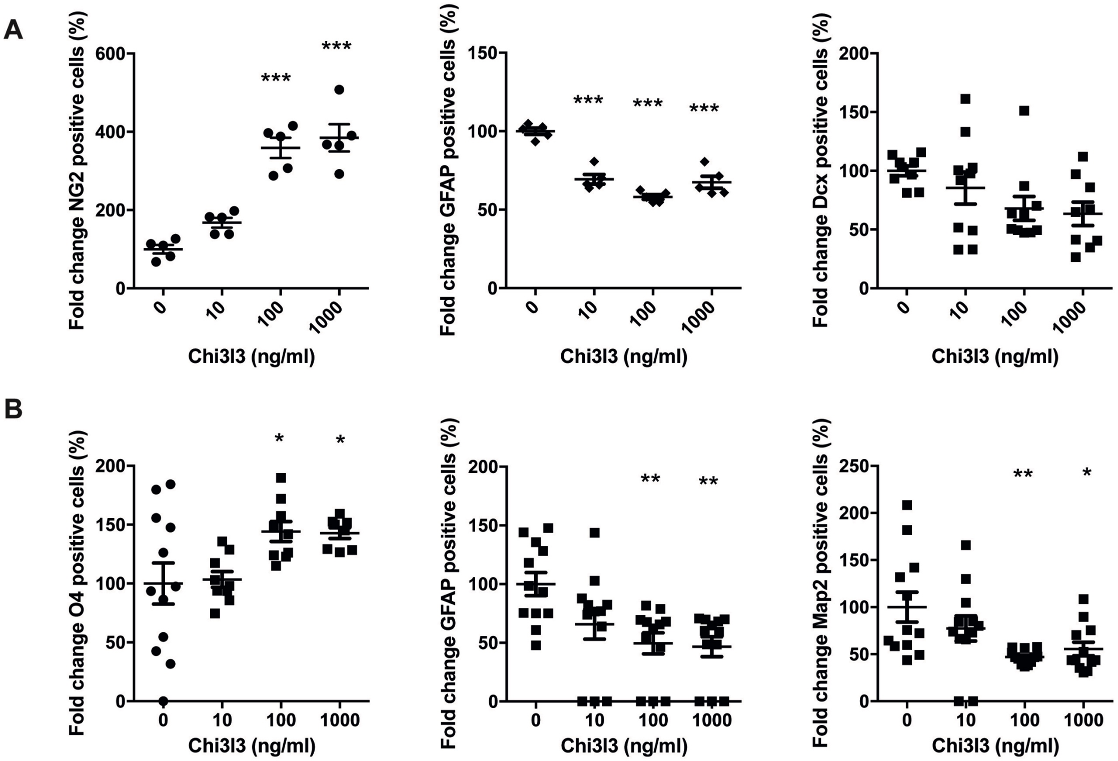

Supplementary Fig. 3. Dose dependency of Chi3l3-modulated lineage decision of neural stem cells *in vitro.*

Quantification of neural stem cells (NCSs) cultured in the presence of increasing concentration of Chi3l3 (0-1000 ng/ml) for 3 days (**A**) or 5 days (**B**). Cells were immunostained for the progenitor lineage markers NG2 (oligodendrocyte precursor cells), GFAP (astrocytes) and doublecortin (Dcx, neural progenitor cells) (**A**) or the mature lineage markers O4 (oligodendrocytes), GFAP (astrocytes) and microtubule-associated protein 2 (Map2, neurons, green) (**B**) and the nuclear stain TO-PRO-3. Quantification is displayed as percentage of marker positive cells of TO-PRO-3 positive cells. Exposure to Chi3l3 during NSC differentiation led to a significant increase in the percentage of $NG2^+$ OPCs and mature $O4^+$ oligodendrocytes and a significant decrease in $GFAP^+$ astrocytes and Dcx^+ neuroblasts and $Map2^+$ neurons in a dose-dependent manner. (one-way ANOVA with Dunnett's post-hoc test; Data are representative of 3 independent experiments with the following number of replicates: (A, NG2 & GFAP) $n = 5$; (A, Dcx) $n = 9$ (0, 1000) and $n = 10$ (10, 100); (B, O4) $n = 12$ (0), $n = 9$ (10, 100) and $n = 8$ (1000); (B, GFAP, Map2) $n = 12$; mean ± s.e.m *p < 0.05; **p < 0.01; ***p < 0.005).

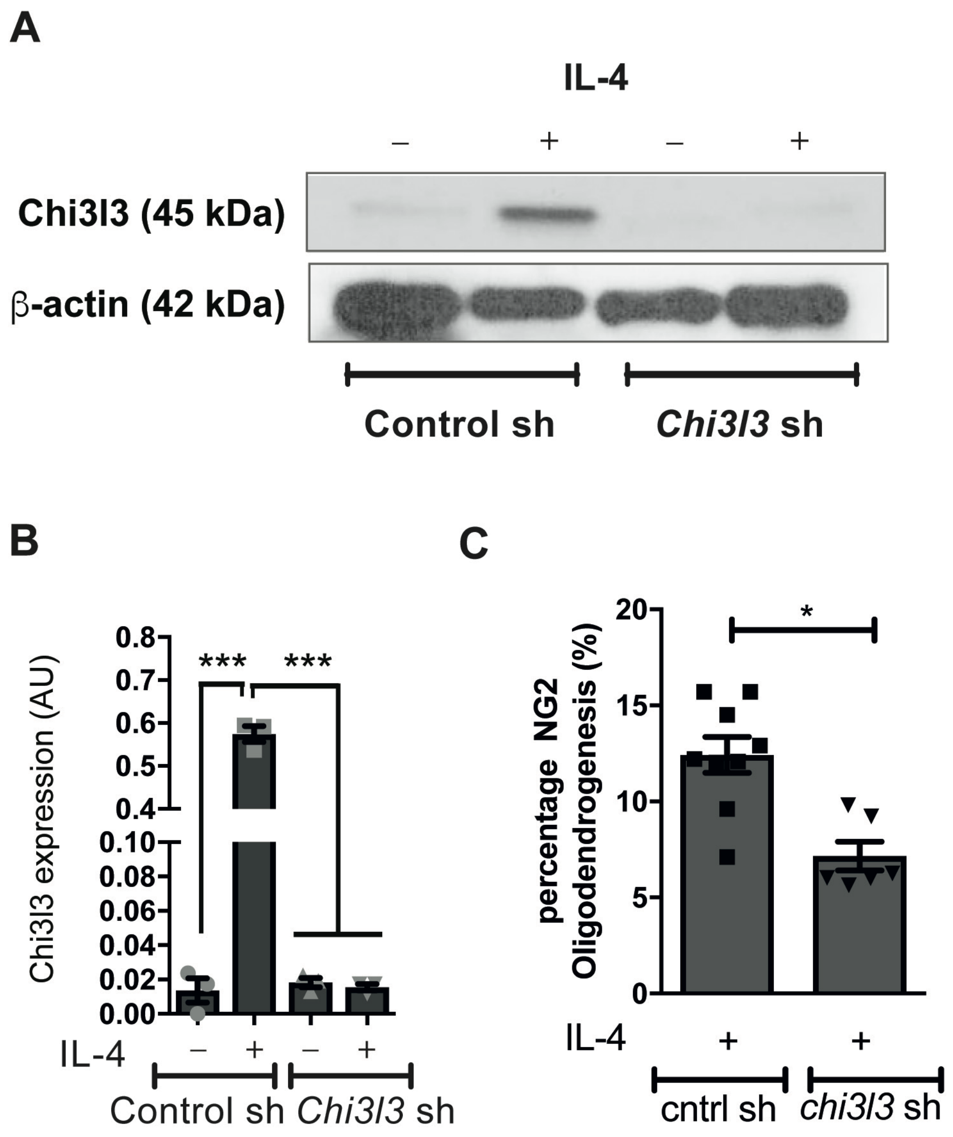

Supplementary Fig. 4. Chi3l3 is required for microglia-mediated oligodendrogenesis.
Western blot analysis (**A**) and quantification of relative Chi3l3 expression (**B**) of BV-2 microglia treated with LVPs containing Chi3l3 shRNA (Chi3l3 sh) or control shRNA (control sh) and stimulated with IL-4. Chi3l3 was significantly reduced in Chi3l3 sh-treated microglia after stimulation with IL-4. No Chi3l3 was detected in unstimulated microglia of both groups. Data is expressed in arbitrary units (AU). one-way ANOVA with Turkey's post-hoc test, Data are representative of 3 independent experiments with *n* = 3 replicates.
(**C**) Percentage of $NG2^+$ oligodendrocyte precursor cells (OPCs) newly generated from neural stem cells differentiated in conditioned medium of control sh or Chi3l3 sh-treated microglia stimulated with IL-4. Knockdown of *Chi3l3* expression led to significant reduction of baseline and IL-4 induced microglia-mediated oligodendrogenesis (two-tailed Student's t-test; Data are representative of 3 independent experiments with *n* = 9 (Control sh / IL4); *n* = 6 (Chi3l3 sh / IL4); mean ± s.e.m *p < 0.05; **p < 0.01; ***p < 0.005.)

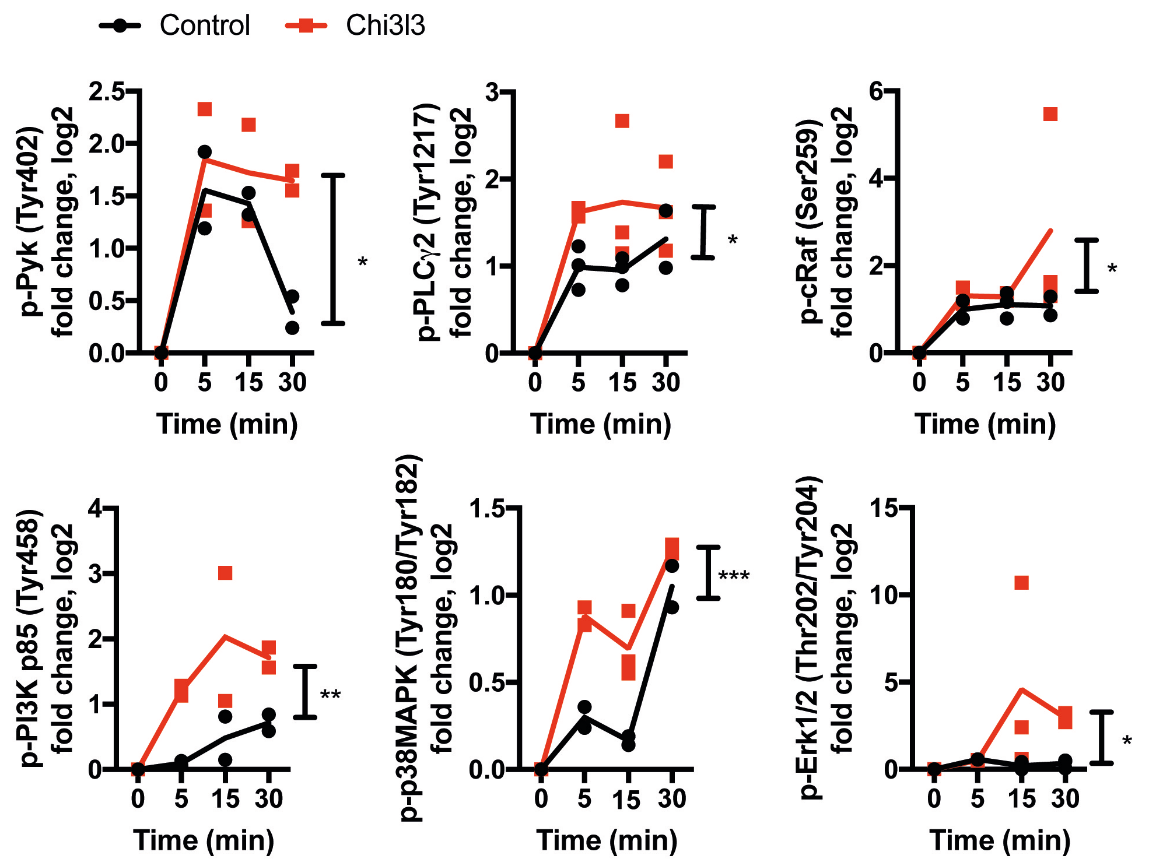

Supplementary Fig. 5. Signaling events mediating Chi3l3-induced activation of neural stem cells *in vitro*

Signaling pathways activated in neural stem cells by Chi3l3 or PBS (control), lysed at 5, 15 and 30 min. Signaling-transduction arrays prepared with lysates were probed with phosphorylation-specific antibodies [1,2]. Phosphorylated (p-) residues are in parentheses on vertical axis. (n = 2; two-way ANOVA; mean ± s.e.m.; *p < 0.05; **p < 0.01; ***p < 0.005).

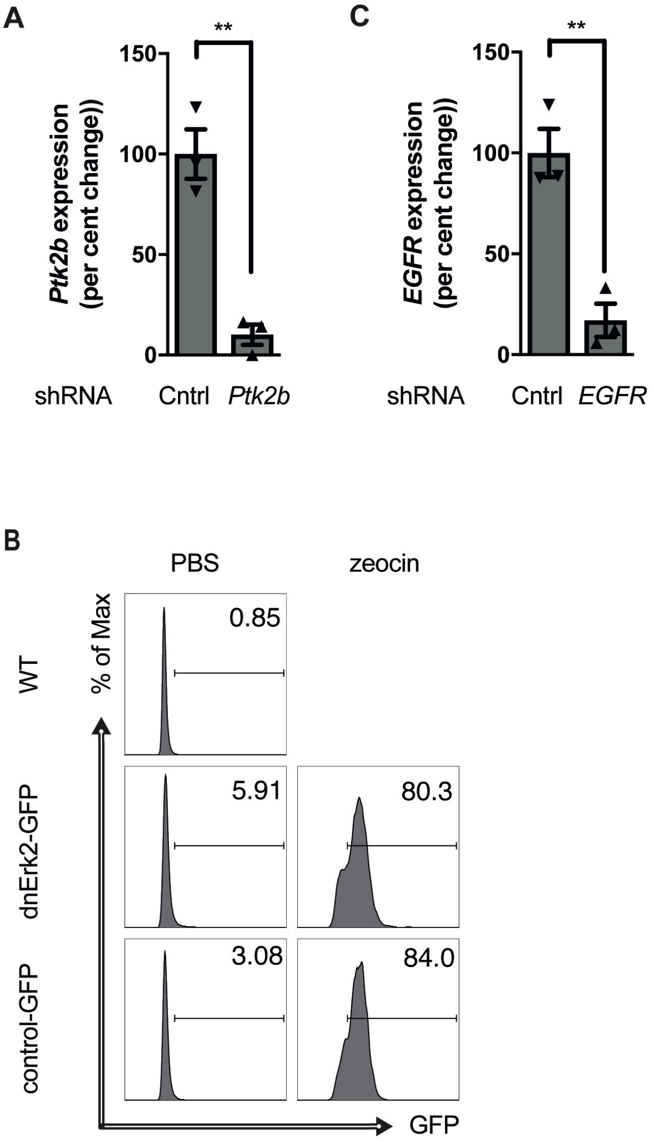

Supplementary Fig. 6. Generation of signaling deficient NSCs
(**A**) *Ptk2b* expression in neural stem cells (NSCs) stably transected with shRNA against *Ptk2b* (Pyk2) or control shRNA (Cntrl). (**B**) Histogram of flow cytometry analysis of GFP expression of wild type (WT) NSCs and NSCs stably transfected with dnErk2-GFP or control-GFP constructs with and without selection using zeocin (**C**) *EGFR* expression in NSCs stably transfected with shRNA against *EGFR* or control shRNA (Cntrl). two-tailed Student's t-test; mean ± s.e.m; Data are representative of 3 independent experiments with $n = 3$ replicates (A,C) (**p < 0.01).

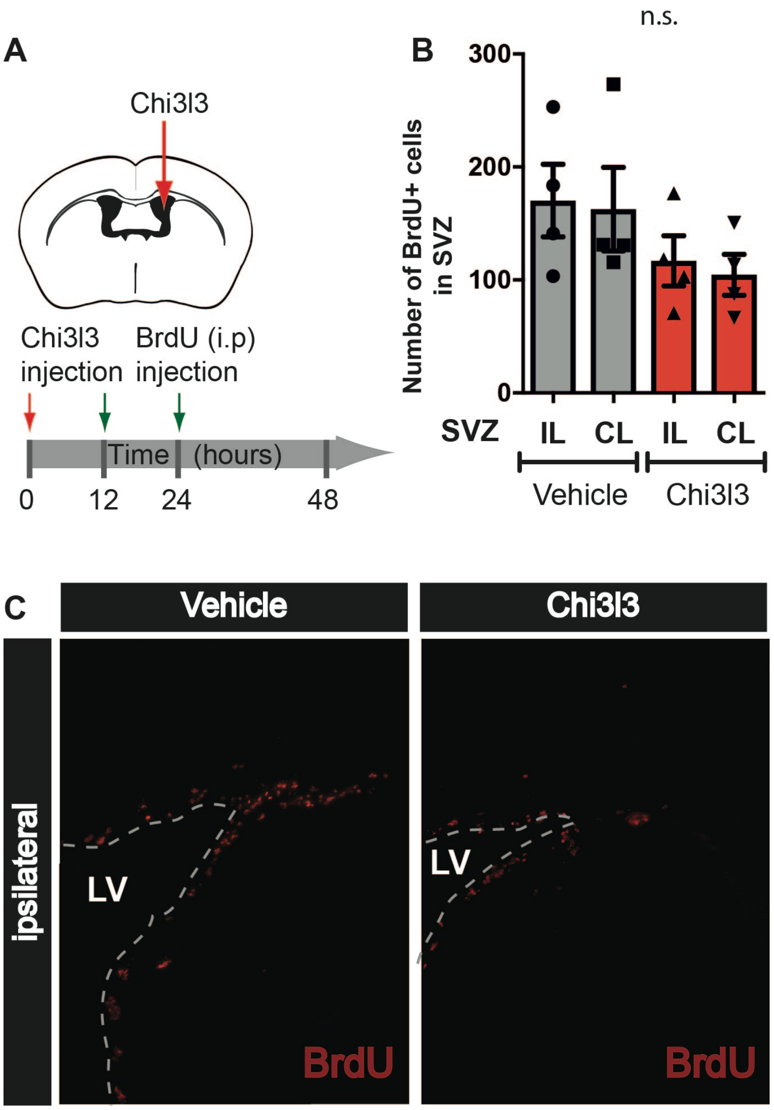

Supplementary Fig. 7. Intraventricular Chi3l3 infusion does not affect proliferation of subventricular zone neural stem cells *in vivo.*

(**A**) Protocol of BrdU labeling via intraperitoneal (i.p.) and Chi3l3 administration. (**B**) Quantification and (**C**) representative confocal images of the ipsilateral (IL) subventricular zone (SVZ) of naïve mice infused with PBS (vehicle, left panel) or Chi3l3 (right panel) and immunostained for BrdU (red). Dashed lines mark the ventricular wall of the right lateral ventricle (LV). Quantifications show a slightly, but not significantly lower number of BrdU positive cells in the SVZ of Chi3l3 infused mice (n = 4 mice per group; one-way ANOVA with Turkey's post-hoc test; mean ± s.e.m.; n.s.= not significant).

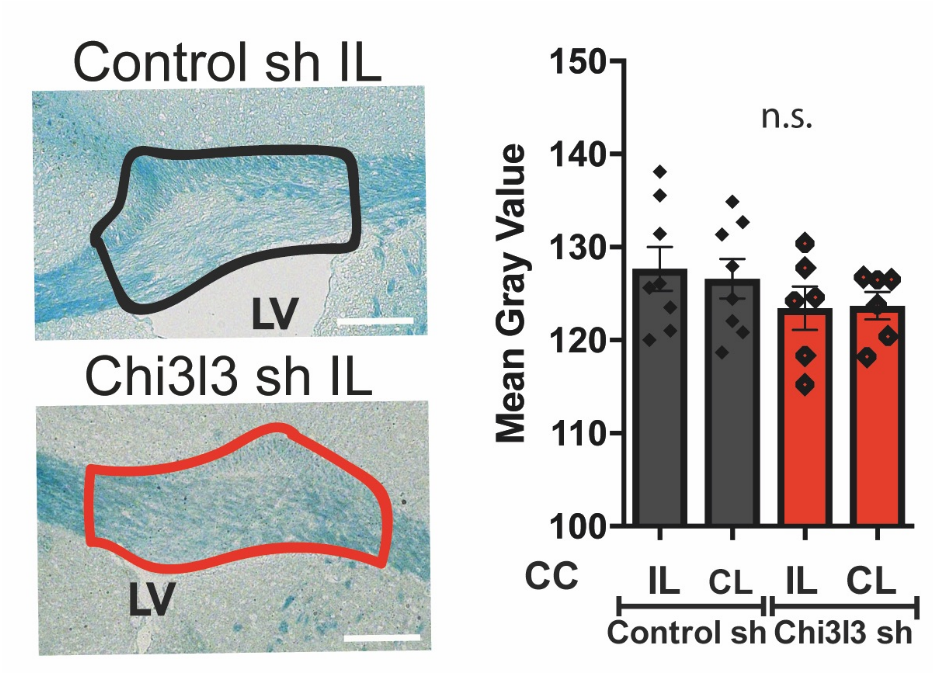

Supplementary Fig. 8 Chi3l3 does not affect total myelin fraction in the corpus callosum of chronic EAE mice

Representative images (left) and quantifications (right) of total myelin stain measured as mean grey value of LFB staining in the ipsilateral (IL) and contralateral (CL) corpus callosum (CC) of Chi3l3 shLVP and control shLVP mice sacrificed during chronic EAE. Low mean gray area values correspond to high levels of myelin staining. (n = 8 mice control shRNA, n = 6 mice Chi3l3 shRNA; one-way ANOVA with Turkey's post-hoc test; mean ± s.e.m; scale bar = 250μm; n.s.=not significant, LV=lateral ventricle).

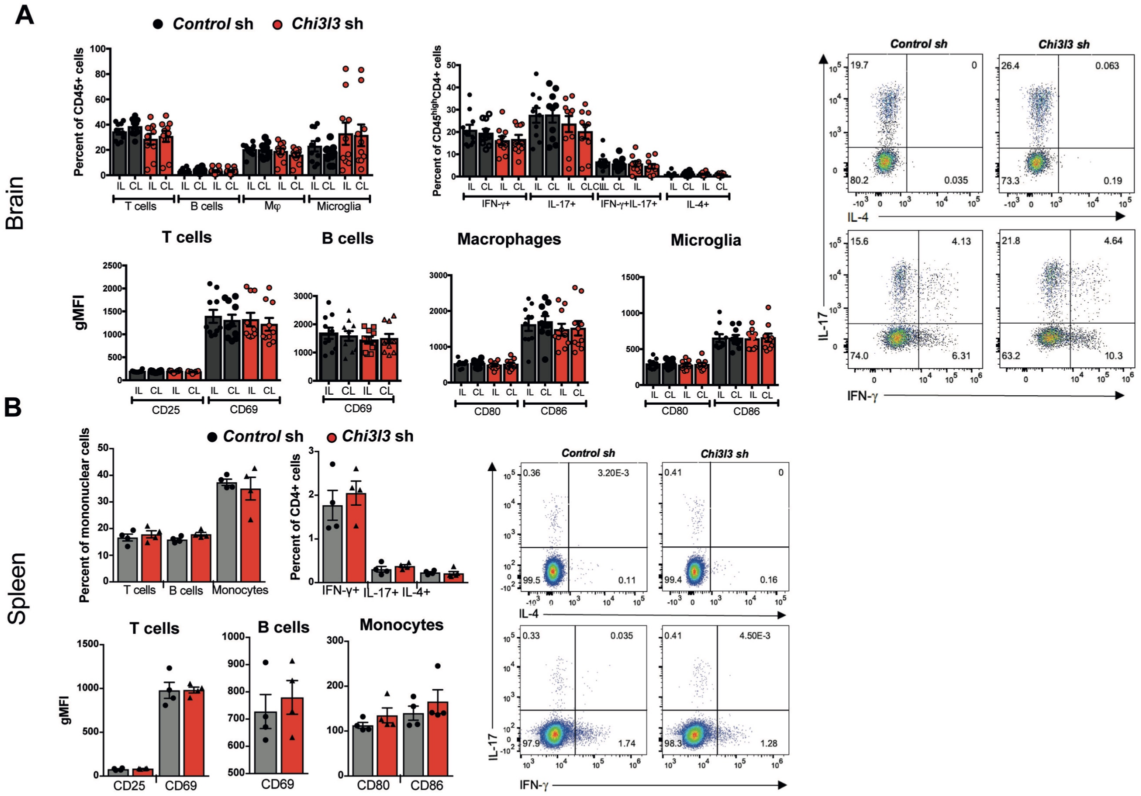

Supplementary Fig. 9 Chi3l3 does not affect immune activity in the brain and periphery at EAE onset. Mice were injected LVPs containing control or Chi3l3 sh RNA in the right ventricle. EAE was induced one week later and the immune cell activation in the brain (**A**) and spleen (**B**) was analyzed at disease onset by flow cytometry. (**A**) The upper left graph shows the percentage of T cells ($CD45^{high}CD4^{+}$), B cells ($CD45^{high}B220^{+}$), macrophages ($CD45^{high}CD11b^{+}$) and microglia ($CD45^{low}CD11b^{+}$) relative to all immune $CD45^{+}$(low/high) cells in each brain hemisphere. The upper right graph shows the percentage of the indicated cytokines in the $CD45^{high}CD4^{+}$ cells. At the right, a representative dot plot is shown. The lower graphs show the gMFI of the indicated activation markers in each cell population. $n = 10$ of two independent experiments. The control and *Chi3l3* sh injected IL hemispheres were analyzed with a two-tailed student t-test; the data was not significantly different ($p<0.05$). IL, ipsilateral; CL, contralateral; Mφ, macrophages; gMFI, geometric mean fluorescence intensity. Graphs show the mean ± s.e.m. (**B**) The upper left graph shows the percentage of T cells ($CD4^{+}$), B cells ($B220^{+}$) and monocytes ($CD11b^{+}$) relative to the total number of mononuclear cells. The upper right graph shows the percentage of the indicated cytokines in the $CD4^{+}$ cells. At the right, a representative dot plot is shown. The lower graphs show the gMFI of the indicated activation markers in each cell population. $n = 4$, the groups were analyzed with a two-tailed student t-test; the data was not significantly different ($p<0.05$). Each dot represents an individual animal; mean ± s.e.m. is shown.

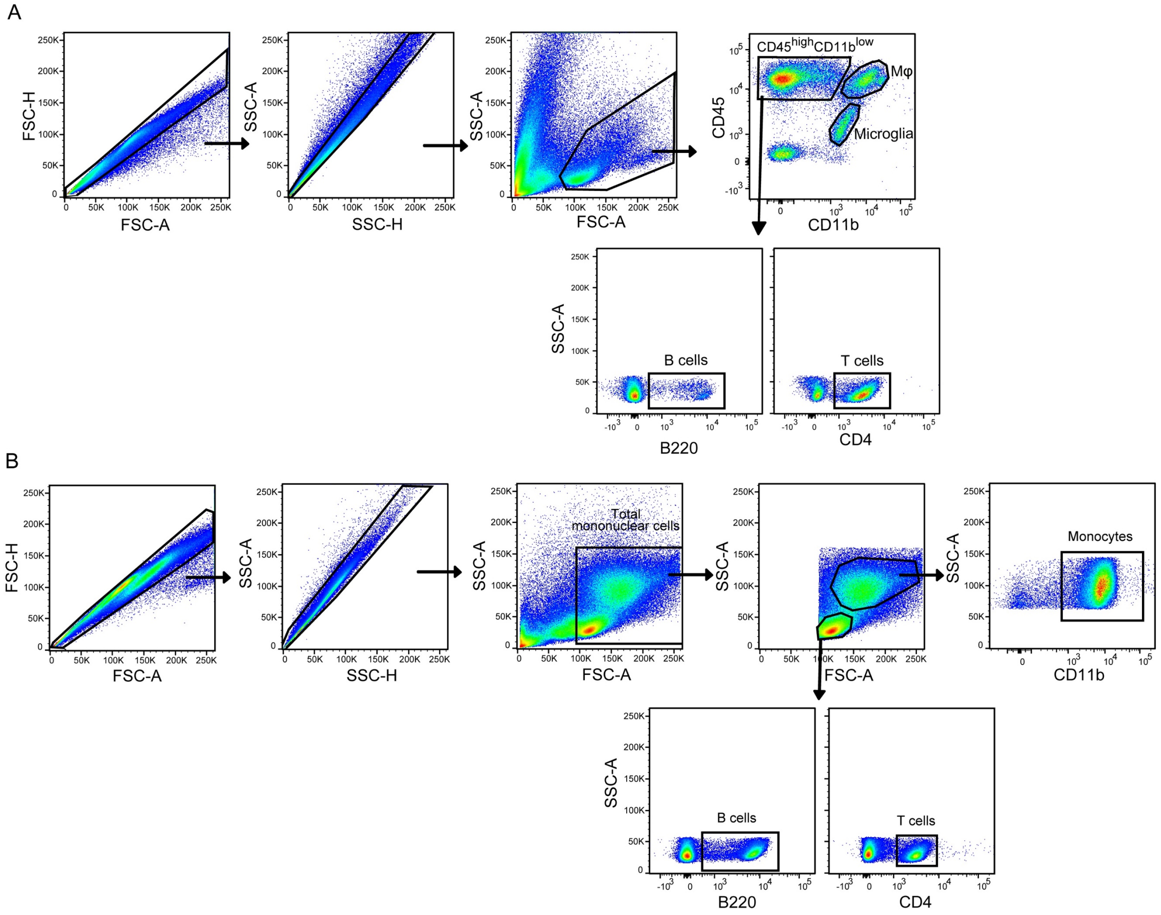

Supplementary Fig. 10 Gating strategy

(**A**) Gating strategy to identify immune populations in the CNS. After discarding duplets, debris and dead cells, macrophages (Mφ) were identified as CD45^{high}CD11b^{high}; microglia as CD45^{low}CD11b^{high}; T cells as CD45^{high}CD11b$^{low/neg}$CD4+ and B cells as CD45^{high}CD11b$^{low/neg}$B220+. (**B**) Gating strategy to identify immune population in the spleen. After discarding duplets, debris and dead cells, monocytes were identified as big and granular CD11b+ cells; T cells and B cells were located in the lymphocyte population as CD4+ and B220+ cells, respectively.

SUPPLEMENTARY METHODS

Reverse-phase protein microarray

NSCs were cultured under differentiation conditions in DMEM:F12 supplemented with 2% B27 (Life Technnologies). Cells were treated for 5, 15, or 30 min with recombinant Chi3l3 (100 ng/ml) or PBS and then lysed with an equal volume of 2x lysis buffer and were "snap-frozen" to prevent any changes in phosphorylation. Cell lysates were transferred to a 384-well plate and spotted onto SuperEpoxy2 slides (Arrayit) with a robotic microarrayer (Genetix) fitted with solid spotting pins. Nonspecific binding on microarrays was blocked with 5% bovine serum albumin, followed by incubation with a panel of primary antibodies as previously described [2]. Arrays were then washed and incubated with HRP-conjugated antibody anti-rabbit IgG (dilution 1/5000; Cell Signaling). To amplify the signal, Opti-4CN kit (Bio-Rad) was used. Then microarrays were incubated with streptavidin Cy5 (1/500; Jackson Immuno Research) and an antibody anti-actin Cy3 (1/100; Abcam). Antibody reactivity was defined by the log2 of the fold change of the mean intensity of binding to the replicates of cell lysates on the microarray.

Western blot analysis

BV-2 cells (carrying control shRNA or a shRNA targeting mouse Chi3l3) were seeded at 25,000 cell/cm^2 density and cultured overnight in neural stem cell differentiation medium. Next day the cells were either stimulated with 20 ng/ml recombinant mouse IL-4 (R&D, 404-ML) for 24 hours or left unstimulated. The cells were lysed in RIPA buffer (Thermo Scientific, 89901) containing 1% protease inhibitor (Thermo Scientific, 78415). The protein concentration of the samples was determined by BCA assay (Thermo Scientific, 23228) according to the manufacturers protocol. 5 μg of total protein from each sample

was loaded on a 10% Bis-Tris gel (Life Technologies, NP0315). The proteins were separated for 1 hour 30 minutes at 150 V and subsequently transferred onto a PVDF membrane at 30 V for 2 hours. The membrane was blocked with 5% nonfat dry milk (BioRad, 170-6404XTU) in TBS-Tween (Thermo Scientific, 28360) for 1 hour and incubated with the following antibodies: rabbit anti mouse Ym1 (Chi3l3) (1:1000, Stemcell Technologies, 01404), mouse anti mouse β-actin (1:5000, Sigma, A5316-2ML). The primary antibodies were detected with corresponding HRP conjugated secondary antibodies (anti-rabbit IgG, Cell Signaling Technologies, 7074P2; anti-mouse IgG, Cell Signaling Technologies; 7076P2; both at a concentration of 1:5000) and the signal was visualized by the means of chemiluminescence (Millipore, P90719). In between incubation with the different primary antibodies the membrane was stripped (Thermo Scientific, 21059) for 15 min and washed four times in TBS-Tween. All primary antibody incubations were carried out overnight at 4°C and all secondary antibodies were incubated for 1 hour at room temperature.

BV-2 microglia conditioned medium transfer assay

BV-2 microglia cells were cultured in microglial culture medium (Dulbecco's modified Eagle's medium with 10% fetal bovine serum), penicillin (50 U/ml), streptomycin (50 mg/ml), sodium pyruvate (1 mM) and L-glutamine (2 mM) at 37°C and 5% CO_2, respectively. Fresh medium was added every 2 days for a total of 7–10 days. Microglial expression of Chi3l3 was silenced by infecting the cells with viral supernatants from 293T cells transfected with Chi3l3 Mission® shRNA plasmid DNA (clone ID: NM_009892.1-567s1c1) or pLKO.1-puro control DNA in a pLKO.1 vector backbone as described above.

For analysis of microglia-mediated oligodendrogenesis, BV-2 cells were cultured in the presence of IL-4 (20 ng/ml, RnD), or PBS (control) for 24h to 48h. Conditioned medium was then transferred to differentiating neural stem cells followed by immunostaining of NG2 and TO-PRO-3 after 3 days of differentiation.

Luxol fast blue staining

Sections were stained with luxol fast blue (LFB) according to standard protocols, and imaged with a light microscope at a magnification of 5X. The mean grey value of the corpus callosum directly above the lateral ventricles was quantified using ImageJ. 2-4 sections per mouse were measured and averaged together.

Flow cytometry analysis of transfected NSCs

For assessment of GFP expression, wildtype, dnErk2-GFP and control GFP cells were measured on a LSRII flow cytometer (BD Bioscience). Compensation and data analysis was performed in FlowJo (version 10.4, Treestar) software. Gating of populations were defined with fluorescence minus one (FMO) staining controls.

Flow cytometry analysis of brain and spleen immune cells

Mice were perfused under anesthesia with cold PBS (without Mg^{+2} and Ca^{+2}), the brain and spleen were extracted and kept in RPMI medium on ice. Each brain hemisphere was processed separately. In brief, the brain tissue was mechanically homogenized with a syringe plug through a 70µm cell strainer (Corning) and washed with RPMI 1640 medium (Gibco) supplemented with 10% fetal calf serum (FCS) and antibiotics. The myelin rich

cell suspension was resuspended in 37% Percoll (Sigma-Aldrich), the myelin-free non-neuronal cells were collected from the pellet after centrifugation at 2800g, and washed two times in medium. Single cell suspension of spleen was obtained by homogenizing the tissue though a 100µm cell strainer; erythrocytes were lysed for 10 minutes with 0.15 M ammonium chloride and washed two times.

Flow cytometry staining was performed at 4°C in 1ml Micronic tubes in PBS without calcium and magnesium (Gibco) containing 0.5% BSA (Serva). First, the cells were incubated for 15 minutes with anti-mouse CD16/CD32 (clone 2.4G2, BD Biosciences) to block the Fc-Receptors. Afterwards, a cocktail of monoclonal anti-mouse antibodies were added and incubated for 20 minutes. For intracellular staining, the FoxP3 staining buffer set (Invitrogen) was used to fix and permeabilize the cells according to the manufacturer's instructions. Intracellular antibodies were incubated for 1 hour at room temperature. For intracellular cytokine detection, half of the cells obtained from each brain hemisphere were incubated with 50 ng/ml of PMA (Sigma-Aldrich), 5µg/ml Ionomycin (Sigma-Aldrich) and 10µg/ml of Brefeldin A (Biolegend) in complete medium (RPMI supplemented with 2 mM L-glutamine (Gibco), 100 U/mL penicillin (Seromed), 100 µg/mL streptomycin (Seromed), 10% FCS (Sigma–Aldrich) and 1% HEPES (Gibco)) in U-bottom 96 well plates, for 5 hours. Splenocytes were incubated for the same time with the same concentration of stimulants in 48 well plates (10^6 cells per well).

The following antibodies were used: Pacific Blue or FITC-conjugated anti-CD45 (clone 30-F11, Biolegend 103125, Invitrogen 11-0451-85), APCCy7-conjugated anti-CD11b (clone M1/70, BD Biosciences 557657), PerCP-conjugated anti-CD4 (clone RM4-5, BD Biosciences 553052), Pacific Blue-conjugated anti-CD3 (clone 500A2, BD Biosciences

558214), APC-conjugated anti-CD25 (clone PC61, BD Biosciences 557192), PE-conjugated anti-CD69 (clone H1.2F3, BD Biosciences 553237), FITC-conjugated anti-CD80 (clone 16-10A71, BD Biosciences 553768), PECy7-conjugates anti-CD86 (clone GL-1, Biolegend 105014), Alexa Fluor 700-conjugated anti-BB20 (clone RA3-6B2, Biolegend 103231), PE-conjugated anti-IFNγ (clone XMG1.2, BD Biosciences 554412), APC-conjugated anti-IL17 (clone eBio17B7, Invitrogen 17-7177-81) and PeCy7-conjugated anti-IL4 (clone 11B11, Biolegend 504117).

Sample acquisition was performed in a BD Fortessa Flow Cytometer (BD Biosciences). Compensation and data analysis was performed in FlowJo (version 10.4, Treestar) software. Gating of populations were defined with fluorescence minus one (FMO) staining controls.

In the brain, populations were defined as follows: Microglia, $CD45^{low}$CD11b+ monocytes/macrophages, $CD45^{high}$CD11b+; B cells, $CD45^{high}$B220+ and T cells $CD45^{high}$CD3+CD4+. In the spleen, populations were located in the forward vs sideward scatter plots by their distinctive and identified by their lineage markers: Monocytes, CD11b+; B cells, B220+ and T cells CD3+CD4+.

1. Kutzelnigg, A. *et al.* Cortical demyelination and diffuse white matter injury in multiple sclerosis. *Brain* **128,** 2705–2712 (2005).
2. Farez, M. F. *et al.* Toll-like receptor 2 and poly(ADP-ribose) polymerase 1 promote central nervous system neuroinflammation in progressive EAE. *Nature Immunology* **10,** 958–964 (2009).
