# Supplementary material for: Chi3l3 induces oligodendrogenesis in an experimental model of autoimmune neuroinflammation
Source: Nat Commun. 2019 Jan 15;10:217. doi: 10.1038/s41467-018-08140-7 (PMC6333780; doi:10.1038/s41467-018-08140-7)
Supplement: Supplementary file 2 — Reporting Summary [file 41467_2018_8140_MOESM2_ESM.pdf]

nature research

Corresponding author(s): Sarah C. Starossom

Reporting Summary

Nature Research wishes to improve the reproducibility of the work that we publish. This form provides structure for consistency and transparency in reporting. For further information on Nature Research policies, see Authors & Referees and the Editorial Policy Checklist.

Statistical parameters

When statistical analyses are reported, confirm that the following items are present in the relevant location (e.g. figure legend, table legend, main text, or Methods section).

n/a	Confirmed	
☐	☒	The exact sample size (*n*) for each experimental group/condition, given as a discrete number and unit of measurement
☐	☒	An indication of whether measurements were taken from distinct samples or whether the same sample was measured repeatedly
☐	☒	The statistical test(s) used AND whether they are one- or two-sided<br>*Only common tests should be described solely by name; describe more complex techniques in the Methods section.*
☐	☒	A description of all covariates tested
☐	☒	A description of any assumptions or corrections, such as tests of normality and adjustment for multiple comparisons
☐	☒	A full description of the statistics including central tendency (e.g. means) or other basic estimates (e.g. regression coefficient) AND variation (e.g. standard deviation) or associated estimates of uncertainty (e.g. confidence intervals)
☐	☒	For null hypothesis testing, the test statistic (e.g. *F*, *t*, *r*) with confidence intervals, effect sizes, degrees of freedom and *P* value noted<br>*Give P values as exact values whenever suitable.*
☒	☐	For Bayesian analysis, information on the choice of priors and Markov chain Monte Carlo settings
☐	☒	For hierarchical and complex designs, identification of the appropriate level for tests and full reporting of outcomes
☒	☐	Estimates of effect sizes (e.g. Cohen's *d*, Pearson's *r*), indicating how they were calculated
☐	☒	Clearly defined error bars<br>*State explicitly what error bars represent (e.g. SD, SE, CI)*

Our web collection on statistics for biologists may be useful.

Software and code

Policy information about availability of computer code

Data collection	n/a
Data analysis	n/a

For manuscripts utilizing custom algorithms or software that are central to the research but not yet described in published literature, software must be made available to editors/reviewers upon request. We strongly encourage code deposition in a community repository (e.g. GitHub). See the Nature Research guidelines for submitting code & software for further information.

Data

Policy information about availability of data

All manuscripts must include a data availability statement. This statement should provide the following information, where applicable:

- Accession codes, unique identifiers, or web links for publicly available datasets
- A list of figures that have associated raw data
- A description of any restrictions on data availability

The data that support the findings of this study are available from the corresponding author upon reasonable request.

Field-specific reporting

Please select the best fit for your research. If you are not sure, read the appropriate sections before making your selection.

☒ Life sciences ☐ Behavioural & social sciences ☐ Ecological, evolutionary & environmental sciences

For a reference copy of the document with all sections, see nature.com/authors/policies/ReportingSummary-flat.pdf

Life sciences study design

All studies must disclose on these points even when the disclosure is negative.

Sample size	No sample size calculation was performed. Sample size was chosen based on previous experiments and significance levels determined as described in the individual Figure legends.
Data exclusions	No data were excluded.
Replication	In vitro experiments were repeated at least two times with at least n=3 independent technical replicates or three times with at least n=2 independent technical replicates. In vivo experiments included at least n=3 mice. Individual repeats and sample sizes as well as significance levels are indicated in the Figure legends; statistical significance was determined as described in Figure legends.
Randomization	The in vitro experiments were not randomized. For in vivo experiments, mice were randomly subjected to each group.
Blinding	The investigators were blinded as to experimental groups during data collection and analysis. Specifically, for in vivo experiments, EAE scoring and subsequent tissue analysis was performed by an independent researcher, who was blinded to experimental treatment groups.

Reporting for specific materials, systems and methods

Materials & experimental systems

n/a	Involved in the study	
☒	☐	Unique biological materials
☐	☒	Antibodies
☐	☒	Eukaryotic cell lines
☒	☐	Palaeontology
☐	☒	Animals and other organisms
☒	☐	Human research participants

Methods

n/a	Involved in the study	
☒	☐	ChIP-seq
☐	☒	Flow cytometry
☒	☐	MRI-based neuroimaging

Antibodies

Antibodies used

Immunohistochemistry:
primary antibodies:
rat anti-BrdU (1:100; Accurate Chemical, cat# YSRTMCA2060GA), rat anti-CD11b (1:50; BD Biosciences, cat# 550282), mouse anti-CD45 (1:100, BioLegend, cat# 103101), rat anti-CD4 (1:200, BD Bioscience, cat# 550278) rabbit-anti Chi3l3 (1:50; Stemcell Technology, cat# 60130), rabbit anti-Doublecortin (Dcx, 1:100; Abcam, cat# ab18723), mouse anti-GFAP (1:500; BD Bioscience, cat# 610566), mouse anti-Ki76-FITC (1:100; BD Bioscience, cat# 617472), mouse anti-Map2 (1:250; Sigma-Aldrich, cat# M9942), rabbit anti-NG2 (1:100; Millipore, cat# ab5320), mouse anti-O4 (1:100; Millipore, MAB345), rabbit anti-p-Pyk2 (Tyr402; 1:500, CST, cat# 3291S), rabbit anti-p-cRaf (Ser259; 1:500, CST, cat# 9421P), rabbit anti-Sox2 (1:500, Thermo Fisher Scientific, cat# A24339), rabbit anti-p-p38MAPK (THr180/Tyr182; 1:500, CST, cat#4511P), rabbit anti-p-PLCγ2 (Tyr1217; 1:500, CST, cat# 3871P), rabbit anti-p-PI(3)K (Tyr458; 1:500, CST cat# 4228P), rabbit anti-p-Erk1/2 (Thr202/Tyr204; 1:500, CST, cat# 9101), rabbit anti-p-EGFR (Tyr1068; 1:100, CST, cat# 3777). rat anti-MBP (1: 125, Millipore, cat# MAB386), goat anti-DCX (1:250, SantaCruz, cat# sc-8066), goat anti-SOX2 (1: 250, R&D Systems, cat# AF2018), goat anti-GFAP (1: 250, Santa Cruz, cat# sc-6170), rabbit anti-NG2 (1: 125, Millipore, cat# AB5320)
secondary antibodies:
Alexa Fluor 488 donkey anti-rat IgG (1:250, Thermofisher scientific, A21208), Alexa Fluor 488 donkey anti-rabbit IgG (1:250, Thermofisher scientific, A21206), Alexa Fluor 594 donkey anti-goat IgG (1:250, Thermofisher scientific, A11058)

reverse phase protein microarray:
primary antibodies: for the complete list please refer to Suppl. Table 6 from Farez et al Nat. Immunol 2009

Western blot:

primary antibodies: rabbit anti mouse Ym1 (Chi3l3) (1:1000, Stemcell Technologies, 01404), mouse anti mouse β-actin (1:5000, Sigma, A5316-2ML)
secondary antibodies:
anti-rabbit IgG, Cell Signaling Technologies, 7074P2; anti-mouse IgG, Cell Signaling Technologies; 7076P2; both at a concentration of 1:5000

FACS:
Pacific Blue or FITC-conjugated anti-CD45 (clone 30-F11, Biolegend 103125, Invitrogen 11-0451-85), APCCy7-conjugated anti-CD11b (clone M1/70, BD Biosciences 557657), PerCP-conjugated anti-CD4 (clone RM4-5, BD Biosciences 553052), Pacific Blue-conjugated anti-CD3 (clone 500A2, BD Biosciences 558214), APC-conjugated anti-CD25 (clone PC61, BD Biosciences 557192), PE-conjugated anti-CD69 (clone H1.2F3, BD Biosciences 553237), FITC-conjugated anti-CD80 (clone 16-10A71, BD Biosciences 553768), PECy7-conjugates anti-CD86 (clone GL-1, Biolegend 105014), Alexa Fluor 700-conjugated anti-BB20 (clone RA3-6B2, Biolegend 103231), PE-conjugated anti-IFNγ (clone XMG1.2, BD Biosciences 554412), APC-conjugated anti-IL17 (clone eBio17B7, Invitrogen 17-7177-81) and PeCy7-conjugated anti-IL4 (clone 11B11, Biolegend 504117)

Validation

Describe the validation of each primary antibody for the species and application, noting any validation statements on the manufacturer's website, relevant citations, antibody profiles in online databases, or data provided in the manuscript.

Eukaryotic cell lines

Policy information about cell lines

Cell line source(s)

- 293T cells were purchased from Life Technologies.
- H9 hESC derived NSCs (H9, N7800-100) were purchased from Thermofisher scientific. .
- BV2 cells were acquired as a kind gift from F. Aloisi (Instituto Superiore di Sanità, 00161 Rome, Italy.

Authentication

- 293T cells were authenticated by Life Technologies.
- H9 hESC derived NSCs (H9, N7800-100) were authenticated by the vendor and additional human genome sequencing and microarray analysis was performed (data not shown)
- BV2 cells were positively tested for microglial markers, however no further authentication was performed.

Mycoplasma contamination

Testing for mycoplasma contamination was not performed.

Commonly misidentified lines
(See ICLAC register)

n/a

Animals and other organisms

Policy information about studies involving animals; ARRIVE guidelines recommended for reporting animal research

Laboratory animals

Female SJL/J/6 mice were purchased from the Jackson Laboratory (Bar Harbor, ME, USA). All animals were housed in pathogen-free facilities. All experiments were performed with the approval of the local animal welfare committees (Harvard Medical Area Standing Committee on Animals, and LAGeSo, Berlin) and in accordance with national and international guidelines to minimize discomfort to animals (NIH and 86/609/EEC).

Wild animals

are not included

Field-collected samples

was not performed.

Flow Cytometry

Plots

Confirm that:

☒ The axis labels state the marker and fluorochrome used (e.g. CD4-FITC).

☒ The axis scales are clearly visible. Include numbers along axes only for bottom left plot of group (a 'group' is an analysis of identical markers).

☒ All plots are contour plots with outliers or pseudocolor plots.

☒ A numerical value for number of cells or percentage (with statistics) is provided.

Methodology

Sample preparation

Mice were perfused under anesthesia with cold PBS (without Mg+2 and Ca+2), the brain and spleen were extracted and kept in RPMI medium on ice. Each brain hemisphere was processed separately. In brief, the brain tissue was mechanically homogenized with a syringe plug through a 70µm cell strainer (Corning) and washed with RPMI 1640 medium (Gibco) supplemented with 10% fetal calf serum (FCS) and antibiotics. The myelin rich cell suspension was resuspended in 37% Percoll (Sigma-Aldrich), the myelin-free non-neuronal cells were collected from the pellet after centrifugation at 2800g, and washed two times in medium. Single cell suspension of spleen was obtained by homogenizing the tissue though a 100µm cell strainer; erythrocytes were lysed for 10 minutes with 0.15 M ammonium chloride and washed two times.
Flow cytometry staining was performed at 4°C in 1ml Micronic tubes in PBS without calcium and magnesium (Gibco) containing 0.5% BSA (Serva). First, the cells were incubated for 15 minutes with anti-mouse CD16/CD32 (clone 2.4G2, BD Biosciences) to

block the Fc-Receptors. Afterwards, a cocktail of monoclonal anti-mouse antibodies were added and incubated for 20 minutes. For intracellular staining, the FoxP3 staining buffer set (Invitrogen) was used to fix and permeabilize the cells according to the manufacturer's instructions. Intracellular antibodies were incubated for 1 hour at room temperature. For intracellular cytokine detection, half of the cells obtained from each brain hemisphere were incubated with 50 ng/ml of PMA (Sigma-Aldrich), 5µg/ml Ionomycin (Sigma-Aldrich) and 10µg/ml of Brefeldin A (Biolegend) in complete medium (RPMI supplemented with 2 mM L-glutamine (Gibco), 100 U/mL penicillin (Seromed), 100 µg/mL streptomycin (Seromed), 10% FCS (Sigma–Aldrich) and 1% HEPES (Gibco)) in U-bottom 96 well plates, for 5 hours. Splenocytes were incubated for the same time with the same concentration of stimulants in 48 well plates (106 cells per well).

Instrument

Sample acquisition was performed in a BD Fortessa Flow Cytometer (BD Biosciences) or a LSRII (BD Biosciences)

Software

Compensation and data analysis was performed in FlowJo (version 10.4, Treestar) software.

Cell population abundance

n/a

Gating strategy

Gating of populations were defined with fluorescence minus one (FMO) staining controls.

☒ Tick this box to confirm that a figure exemplifying the gating strategy is provided in the Supplementary Information.
